# Two distinct SNARE complexes mediate vesicle fusion with the plasma membrane to ensure effective development and pathogenesis of *Fusarium oxysporum* f. sp. *cubense*

**DOI:** 10.1111/mpp.13443

**Published:** 2024-03-19

**Authors:** Zhenyu Fang, Qiwen Zhao, Shiyu Yang, Yan Cai, Wenqin Fang, Yakubu Saddeeq Abubakar, Ying Lin, Yingzi Yun, Wenhui Zheng

**Affiliations:** ^1^ State Key Laboratory of Ecological Pest Control for Fujian and Taiwan Crops, College of Plant Protection Fujian Agriculture and Forestry University Fuzhou Fujian China; ^2^ Key Laboratory of Biopesticide and Chemical Biology, Ministry of Education, College of Plant Protection Fujian Agriculture and Forestry University Fuzhou Fujian China; ^3^ Department of Biochemistry, Faculty of Life Sciences Ahmadu Bello University Zaria Nigeria; ^4^ School of Biological and Environmental Engineering Jingdezhen University Jingdezhen Jiangxi China

**Keywords:** banana Fusarium wilt fungus, exocytosis, pathogenicity, SNARE, vesicle

## Abstract

SNAREs (soluble *N*‐ethylmaleimide‐sensitive factor attachment protein receptors) facilitate docking and fusion of vesicles with their target membranes, playing a crucial role in vesicle trafficking and exocytosis. However, the spatial assembly and roles of plasma membrane (PM)‐associated SNAREs in phytopathogen development and pathogenicity are not clearly understood. In this study, we analysed the roles and molecular mechanisms of PM‐associated SNARE complexes in the banana Fusarium wilt fungus *Fusarium oxysporum* f. sp. *cubense* tropical race 4 (FocTR4). Our findings demonstrate that FocSso1 is important for the fungal growth, conidiation, host penetration and colonization. Mechanistically, FocSso1 regulates protein secretion by mediating vesicle docking and fusion with the PM and hyphal apex. Interestingly, a FocSso1–FocSec9–FocSnc1 complex was observed to assemble not only at the fungal PM but also on the growing hyphal apex, facilitating exocytosis. FocSso2, a paralogue of FocSso1, was also found to form a ternary SNARE complex with FocSec9 and FocSnc1, but it mainly localizes to the PM in old hyphae. The functional analysis of this protein demonstrated that it is dispensable for the fungal growth but necessary for host penetration and colonization. The other subunits, FocSec9 and FocSnc1, are involved in the fungal development and facilitate host penetration. Furthermore, FocSso1 and FocSnc1 are functionally interdependent, as loss of FocSso1 leads to mis‐sorting and degradation of FocSnc1 in the vacuole and vice versa. Overall, this study provides insight into the formation of two spatially and functionally distinct PM SNARE complexes and their involvement in vesicle exocytosis to regulate development and pathogenicity of FocTR4.

## Graphical abstract

Two SNARE complexes Sso1–Sec9–Snc1 and Sso2–Sec9–Snc1 were identified in *Fusarium oxysporum* f. sp. *cubense* that coordinately facilitate docking and fusion of exocytic vesicles with the plasma membrane in a spatially specific way.

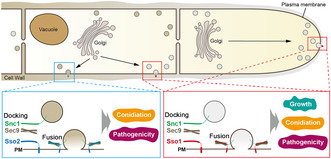

## 1 INTRODUCTION

Transport vesicles play vital roles in numerous cellular processes, including the exchange of material, energy and information between organelles or cells (Abubakar et al., 2023; Shoji et al., 2014). These important functions are achieved through cargo sorting and transportation facilitated by various trafficking machineries (Aniento et al., 2022; Chi et al., 2015; D'Souza et al., 2021). Transport of vesicles to a given destination involves multiple steps, such as the vesicles formation from donor cells, movement of the vesicles, fusion of the vesicles with target membranes and other associated processes (Gerst, 1999). Among the proteins involved in this sequence of events, SNAREs (soluble *N*‐ethylmaleimide‐sensitive factor attachment protein receptors) are particularly emphasized, as they are necessary for the crucial steps in membrane fusion (Gerst, 1999; Stanton & Hughson, 2023).

All SNARE proteins possess an evolutionarily conserved SNARE domain, which spans 60–70 amino acids (Chen & Scheller, 2001; Hong & Lev, 2014). SNAREs are categorized into v‐SNAREs and t‐SNAREs based on their localization on either vesicles or target membranes (Gerst, 1999; Söllner et al., 1993). However, further discoveries unveiled the existence of SNARE proteins other than these two groups on both vesicles and target membranes. As a result, the SNARE proteins were regrouped into Q‐SNAREs and R‐SNAREs, which represent SNARE proteins that contribute a glutamine (Q) or an arginine (R) to form the zero ionic layers of their assembled core complexes, respectively (Fasshauer et al., 1998). Based on their structures, the Q‐SNAREs are further classified into Qa‐SNAREs, Qb‐SNAREs and Qc‐SNAREs (Bock et al., 2001; Kuratsu et al., 2007). The majority of R‐SNAREs function as v‐SNAREs, while most Q‐SNAREs function as t‐SNAREs (Kuratsu et al., 2007). Interaction between Qabc‐SNAREs and R‐SNAREs results in the formation of a trans‐SNARE complex (also referred to as SNAREpin) (Hong & Lev, 2014). This complex consists of four SNARE motifs that assemble as a twisted parallel four‐helix bundle, allowing the opposing membranes to come together and catalyse membrane fusion (Hong & Lev, 2014). In complicated cellular processes, the same kind of SNARE proteins can assemble into multiple SNAREpins, leading to regulation of various fusion events (Hong, 2005; Malsam et al., 2008). Hence, the precise and temporal assembly of a functional SNAREpin is remarkably important for coordinating different membrane‐trafficking pathways.

In *Saccharomyces cerevisiae*, transportation of small vesicles from the Golgi apparatus to the plasma membrane (PM) relies on either Sso1 or Sso2 (both of which are Qa‐SNAREs) (Aalto et al., 1993). Deletion of both *SSO* genes results in accumulation of secretory vesicles and blockage of extracellular protein secretion (Aalto et al., 1993). Thus, both Sso1 and Sso2 proteins are essential for exocytosis. Additionally, Sec9, a protein with two SNARE motifs, acts as both a Qb‐ and a Qc‐SNARE, and is also essential for exocytosis (Brennwald et al., 1994). Exocytosis also involves the participation of either Snc1 or Snc2, two interchangeable proteins serving as R‐SNAREs. The Snc1/2–Sso1/2–Sec9 SNARE complex forms a SNAREpin and functions in the docking and fusion of exocytic vesicles with the PM (Burri & Lithgow, 2004; Strop et al., 2008). Although studies on PM‐associated SNARE complexes in yeast have made significant progress, the nature, composition and functions of similar complexes in filamentous fungi remain unclear.

Evolutionary analysis of SNARE proteins indicated that filamentous fungi possess homologues of the majority of yeast SNAREs (Gupta & Brent Heath, 2002; Kienle et al., 2009). This suggests that members of the SNARE family are highly conserved among fungi. Genome‐wide analyses suggest that there are 24 SNARE genes present in the yeast genome (Burri & Lithgow, 2004). However, filamentous fungi typically contain 18–22 SNARE genes (Chen et al., 2023; Gupta & Brent Heath, 2002; Kienle et al., 2009; Yang et al., 2013). The extended SNARE genes in yeast are a result of whole‐genome duplication within the *Saccharomyces* lineage (Kienle et al., 2009). For instance, several closely related protein pairs, such as Sso1 and Sso2, Snc1 and Snc2, and Sec9 and Spo20, were discovered in yeast (Kienle et al., 2009). In contrast, with the exception of Sso, only single forms of the corresponding orthologues of these proteins exist in filamentous fungi (such as *Neurospora crassa*, *Aspergillus oryzae* and *Verticillium dahliae*) (Gupta & Brent Heath, 2002; Kuratsu et al., 2007; Wang et al., 2018). These evolutionary data suggest that vesicle trafficking pathways in yeast differ to a large extent from those in many filamentous fungi; however, the reason for this disparity is not clearly understood.

Studies have shown that the exocytic SNAREs in many phytopathogens are important for the fungal development and pathogenicity. In the rice blast fungus *Magnaporthe oryzae*, Sso1 and Snc1 both play indispensable roles in invasive hyphal development and effector secretion (Chen et al., 2023; Giraldo et al., 2013). Targeted deletions of the *SSO1* or *SNC1* gene in *M. oryzae* resulted in a significant reduction in pathogenicity on susceptible rice cultivars (Chen et al., 2023; Giraldo et al., 2013). A recent study found that Sso1–Snc1 interaction is important for the development of the biotrophic interface complex and cytoplasmic effector protein secretion during host infection by *M. oryzae* (Guo et al., 2023). In *Gibberella zeae*, the GzSYN1 (Sso1 orthologue) and GzSYN2 (Sso2 orthologue) are both important for the fungal virulence. Compared to the wild type, the virulence of Δ*Gzsyn1* and Δ*Gzsyn*2 mutants on barley heads reduced by 67% and 75%, respectively (Hong et al., 2010). Additionally, Sso2 is important for deoxynivalenol (DON) accumulation both in vitro and in planta (O'Mara et al., 2020). The Snc1 orthologue in *Fusarium graminearum* was also demonstrated to play an important role in the fungal virulence to wheat heads (Zheng et al., 2018). While these components of the exocytic SNARE complex are vital for fungal virulence, the connection between exocytosis and pathogenesis has not been clearly established. There is also limited knowledge on the mechanism of how this complex with two Sso1 paralogues regulates exocytosis and virulence in phytopathogens.

In this study, we set out to investigate the exocytic dynamics and roles of the components of PM‐associated SNARE complexes in the banana Fusarium wilt fungus *Fusarium oxysporum* f. sp. *cubense* tropical race 4 (FocTR4). This fungus causes Fusarium wilt of banana (*Musa* spp.), also known as Panama disease, which is one of the most devastating plant diseases worldwide (Ma et al., 2023; Maryani et al., 2019). FocTR4 affects nearly all banana cultivars, including Cavendish, which accounts for 28% of local consumption as well as 15% of export products (Maryani et al., 2019). Improving our understanding of the functional mechanisms of these proteins in the development and pathogenesis of FocTR4 will facilitate the development of efficient management strategies against this devastating pathogen.

Herein, we found that, in FocTR4, FocSso1 regulates protein secretion by mediating vesicle docking and subsequent fusion with the PM and hyphal apex. We also clearly demonstrated the existence of two distinct PM‐associated SNARE complexes in FocTR4 in particular, and most likely in filamentous fungi as a whole. One is the FocSso1–FocSec9–FocSnc1 complex, acting at the hyphal apex and PM; the other is the FocSso2–FocSec9–FocSnc1 complex, the function of which is limited to the PM of old hyphae. FocSso1 and FocSso2 play distinct functions in the fungal growth, although both are necessary for host penetration and colonization. Based on our data, we conclude that two distinct PM‐associated SNARE complexes exist that mediate distinct protein secretion pathways to ensure effective growth, development and virulence of FocTR4.

## 2 RESULTS

### 2.1 FocSso1 is important for vegetative growth and sporulation of FocTR4

Sso is generally present in all PM‐associated SNARE complexes. To characterize these complexes in FocTR4, we decided to analyse the nature of FocSso1 in the fungus. Phylogenetic analysis revealed that Sso1 orthologues are highly conserved in eukaryotes and they all contain a syntaxin domain (Figure S1A,B). The *FocSSO1* gene (FOIG_01471) is predicted to encode a 333‐amino acid polypeptide that harbours a transmembrane helix at its C‐terminus, behind the syntaxin domain (Figure S1C). To analyse the functions of FocSso1 in FocTR4, we generated *FocSSO1* gene deletion mutants (Δ*Focsso1*) by replacing its open reading frame (ORF) with a hygromycin phosphotransferase gene through homologous recombination (Figure S2A) and confirmed the deletion by PCR and Southern blot analyses (Figure S2B). Compared to the wild type, the vegetative growth of the Δ*Focsso1* mutant was significantly reduced on complete medium (CM) and minimal medium (MM) plates (Figure S3A,B). Also, the quantities of microconidia and macroconidia produced by the Δ*Focsso1* mutant were significantly less than those produced by the wild type FocTR4 (Figure S3C,D). To confirm that the defects observed in the Δ*Focsso1* mutant were due to the absence of the *FocSSO1* gene, we reintroduced a *GFP‐FocSSO1* fusion construct (pFocSSO1::GFP‐FocSSO1) into the mutant and generated a complemented strain (Δ*Focsso1‐C*). All the defects observed in Δ*Focsso1* mutant were restored to the wild‐type level in the Δ*Focsso1‐C* strain (Figure S3). These suggest that FocSso1 is important for vegetative growth and sporulation in FocTR4.

### 2.2 FocSso1 is indispensable for host penetration and colonization

**FIGURE 1:**
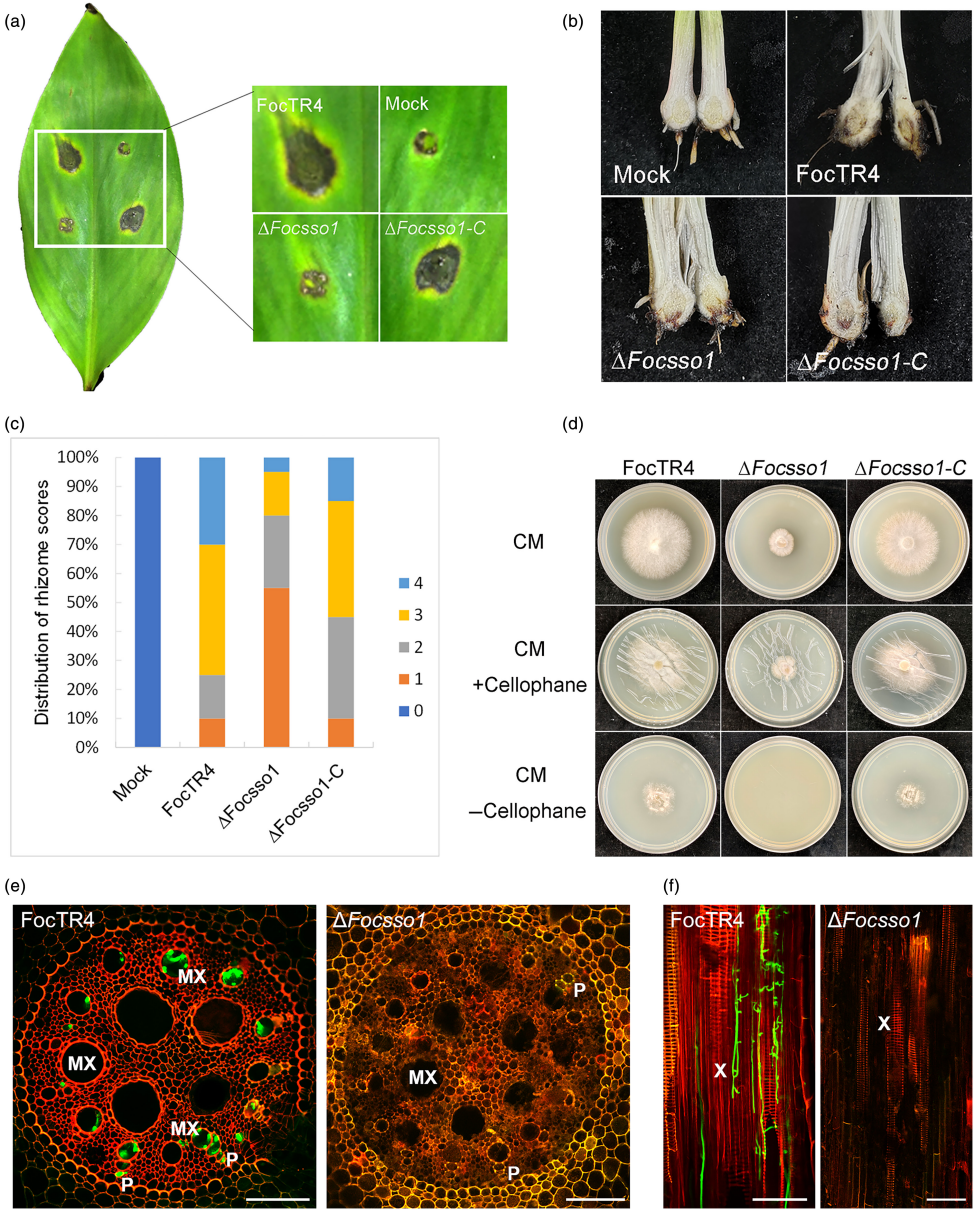
Effect of *FocSSO1* deletion on the virulence of *Fusarium oxysporum* f. sp. *cubense* tropical race 4 (FocTR4). (a) Pathogenicity assay of Δ*Focsso1* mutant on banana leaves. (b) Analysis of ∆*Focsso1* virulence on banana roots. (c) Disease indexes based on the degree of damage inflicted on banana roots. (d) Cellophane penetration ability of the wild type (FocTR4), ∆ *Focsso1* mutant and ∆*Focsso1‐C*. The ∆*Focsso1* mutant was unable to penetrate a single layer of cellophane at 2 days post‐inoculation (dpi). CM, complete medium. (e) Cross sections of banana roots infected with the wild‐type FocTR4 and ∆*Focsso1* mutant at 3 dpi. The presence of invasive hyphae observed in the FocTR4‐infected roots by detection of green fluorescent protein (GFP) signal. This signal could not be detected in the ∆*Focsso1*‐infected banana root, demonstrating the failure of the mutant to colonize the root tissues. MX, metaxylem. P, phloem. Bars, 100 μm. (f) Longitudinal sections of banana roots infected with the wild‐type FocTR4 and ∆*Focsso1* mutant at 3 dpi. GFP signals are similarly detected in the FocTR4‐infected roots but not in the ∆*Focsso1*‐infected ones. X, xylem. Bars, 100 μm.

To further understand the functions of FocSso1 in FocTR4, we evaluated the ability of the Δ*Focsso1* mutant to cause disease symptoms on banana leaves by inoculating the leaves with FocTR4, Δ*Focsso1* mutant and Δ*Focsso1‐C* strains on aseptically wounded spots. The Δ*Focsso1* mutant was only able to develop slight necrotic symptoms on the wounded leaves, and the area of the lesion was obviously less than those of the controls developed under the same incubation conditions (Figure 1a). The mutant necrotic lesion could be due to the effect inflicted by the wound because the mock‐inoculated spot also had similar necrotic lesion (Figure 1a). Similarly, testing the fungal virulence on banana roots revealed that the wild type and the complemented strain caused vascular discolouration on the corms of the banana plantlets. However, the banana plants inoculated with the Δ*Focsso1* mutant had weak necrotic symptoms on the corms (Figure 1b,c), suggesting the involvement of FocSso1 in the virulence of FocTR4.

Cellophane membrane is widely used to study the development of infection structures in plant‐pathogenic fungi (Bourett & Howard, 1990; Zhao et al., 2016). Therefore, to further dissect the link between FocSso1 and the fungal virulence, we investigated the ability of the fungal hyphae to penetrate cellophane membrane and we found that the wild type and the complemented strains were able to penetrate the membrane effectively and establish colonies on CM, while the Δ*Focsso1* mutant failed to form any colony on the medium, suggesting its inability to penetrate the membrane (Figure 1d). This indicates that FocSso1 is indispensable for host penetration. To further confirm this result, we used green fluorescent protein (GFP) as a reporter to trace the presence of the wild type and Δ*Focsso1* mutant hyphae in banana roots after constitutively expressing pRP27::GFP construct in the two strains. At 3 days post‐inoculation (dpi), the hyphae of the GFP‐expressing FocTR4 were clearly found in the vascular tissues of the banana roots (Figure 1e,f). However, the hyphae of the GFP‐expressing Δ*Focsso1* mutant could not be detected in the xylem and phloem tissues of the banana roots by laser confocal microscopy (Figure 1e,f). We conclude that FocSso1 is essential for effective host penetration and pathogenicity in FocTR4.

### 2.3 FocSso1 regulates vesicle docking and fusion at the PM and hyphal apex during protein secretion

**FIGURE 2:**
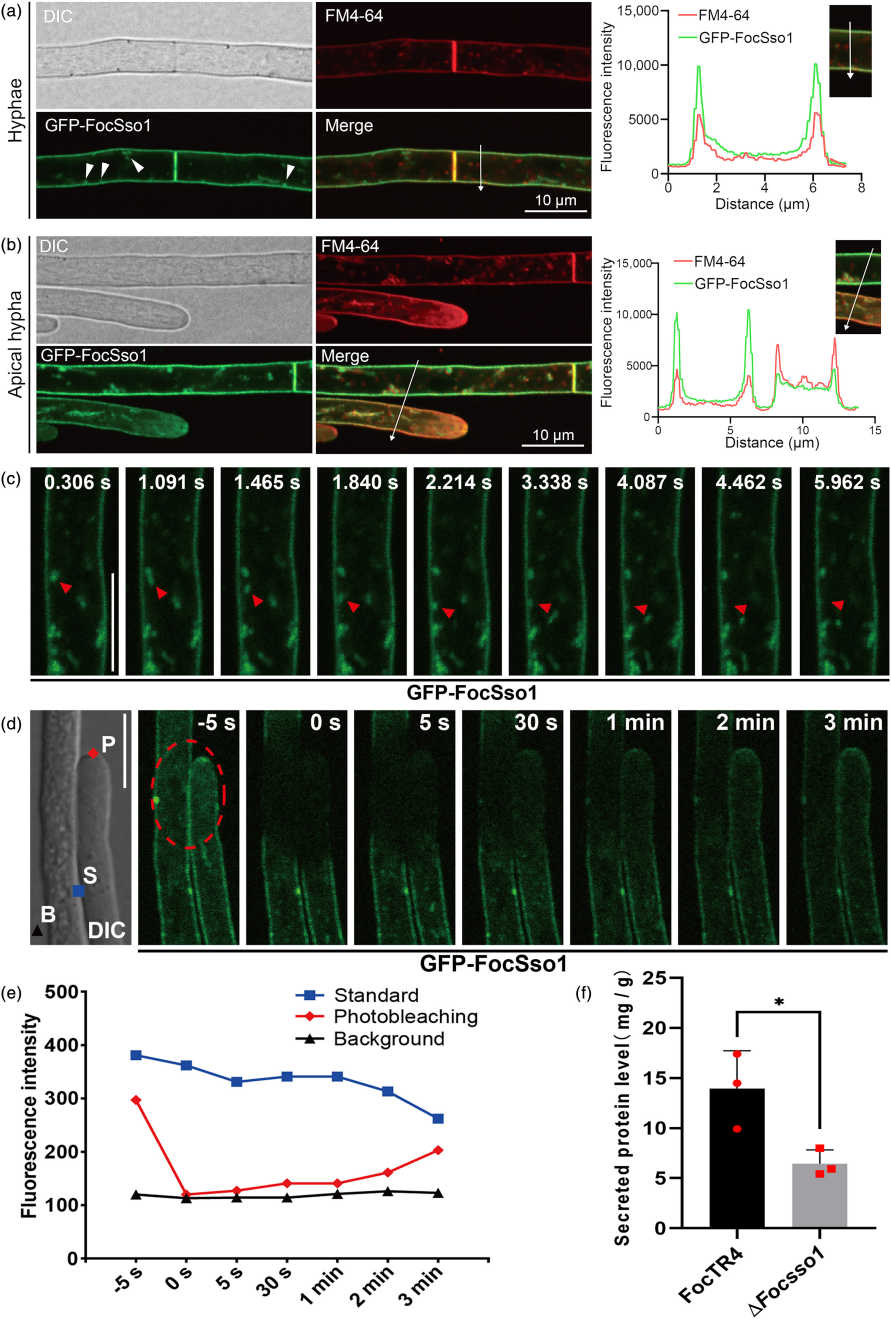
Roles of FocSso1 in protein secretion. (a) Vegetative hyphae expressing GFP‐FocSso1. The hyphae were stained with the endocytic dye FM4‐64 and observed under a confocal microscope. The GFP‐FocSso1 is localized to the plasma membrane and septa, revealed by co‐localization of the GFP signal with FM4‐64. In addition, some GFP‐FocSso1 signals appear in vesicles around the plasma membrane (indicated by white arrowheads). Line graphs were generated at the indicated positions (arrows to show the relative localizations of GFP‐FocSso1 (green) and FM4‐64 (red). DIC, differential interference contrast; GFP, green fluorescent protein. (b) Accumulation of GFP‐FocSso1 at the apical regions of growing hyphae. At the apex, the GFP signal also colocalizes with the FM4‐64‐marked vesicles. (c) Time‐lapse confocal imaging of GFP‐FocSso1 in hyphae. GFP‐FocSso1‐labelled vesicles (red arrowheads) in the hyphae translocate to and/or transiently fused with plasma membrane (Video S1). Bar, 10 μm. (d) Continuous movement of GFP‐FocSso1 to hyphal apex and plasma membrane. Representative time series images of GFP‐FocSso1 on the hyphal apex and plasma membrane immediately after photobleaching (*t* = 0 s). Fluorescence recovery after photobleaching (FRAP) recovery in the photobleaching site after 3 min. Bar, 10 μm. (e) FRAP at the bleaching site relative to the standard and background fluorescence. (f) Amount of proteins secreted by the wild type and Δ*Focsso1* mutant. Values are presented as mean ± *SD* calculated from three independent experiments. *p < 0.05.

For a more in‐depth understanding of the role of FocSso1 in host penetration, we first investigated the subcellular localization of GFP‐FoSso1 in FocTR4. We found that GFP‐FocSso1 mainly localized to the PM, septa and hyphal tips, as evident by its co‐localization with the membrane‐selective dye FM4‐64 (Figure 2a,b). In addition, we also observed GFP‐FocSso1 fluorescence in some mobile vesicles near or attached to the PM (Figure 2a). This prompted us to hypothesize that FocSso1 may be involved in the docking and/or fusion of transport vesicles with the PM during exocytosis. To test this, we first used spinning disk confocal microscopy to track the movement of the GFP‐FocSso1‐labelled vesicles. Our time‐lapse imaging showed that the GFP‐FocSso1‐labelled vesicles moved to the PM and later faded away (Figure 2c, Video S1), implying a likely fusion with the PM. In addition, GFP‐FocSso1‐labelled vesicles were observed to concentrate at the growing hyphal tips, suggesting the involvement of FocSso1 in polarized protein secretion (Video S2). Next, we performed fluorescence recovery after photobleaching (FRAP) analysis to confirm the trafficking of the GFP‐FocSso1‐labelled vesicles to the PM, septa and hyphal apexes. We photobleached the GFP‐FocSso1 in hyphae and then monitored the fluorescence recovery over time (Figure 2c, Figure S4). The analyses revealed fast recovery of the GFP‐FocSso1 fluorescence on the PM, septa and hyphal tips within 3 min (Figure 2d,e, Figure S4), supporting the transport of GFP‐FocSso1 protein to these subcellular locations. To establish the involvement of FocSso1 in protein secretion, we quantified and compared the number of proteins secreted by the wild type and Δ*Focsso1* strains from the supernatant of the fungal liquid cultures. The results showed that the number of proteins secreted to the extracellular space by the Δ*Focsso1* mutant was significantly reduced by about half of that secreted by FocTR4 (Figure 2f). Together, these results indicate that FocSso1 functions in protein secretion by coordinating vesicle docking and fusion with the PM.

### 2.4 FocSso1 negatively regulates the fungal response to cell membrane‐associated stress

Considering that FocSso1 is important for PM‐associated protein secretion, and that protein secretions facilitate adaptation of fungi to environmental stresses on the PM, we decided to investigate the possible role of FocSso1 in tolerance to environmental stresses by growing the various strains on media supplemented with some stress‐inducing agents, including sodium dodecyl sulphate (SDS), NaCl, H_2_O_2_, Congo red (CR) and calcofluor white (CFW). From these assays, we found that Δ*Focsso1* mutant showed increased tolerance to membrane‐associated stresses due to SDS, CR, and CFW, compared to the wild‐type FocTR4 and the complemented strain Δ*Focsso1‐C* (Figure S5A,B). These results show that FocSso1 negatively regulates responses to cell membrane‐associated stresses in FocTR4.

### 2.5 FocSso1 and FocSso2 independently form trimeric SNARE complexes with FocSnc1 and FocSec9

In yeast, Sso proteins (Sso1/Sso2) form ternary SNARE complexes with Snc1/Snc2 (R helix) and Sec9 (Qbc helix) (Brennwald et al., 1994; Rossi et al., 1997). To understand how FocSso1 constitutes a SNARE complex in FocTR4, we screened and identified the interacting partner(s) of GFP‐FocSso1 by immunoprecipitation‐mass spectrometry (IP‐MS) (Figure S6A). By removing the nonspecific interacting partners from the wild‐type (WT)‐GFP, a total of 705 FocSso1‐associated proteins was obtained from the IP‐MS data (Figure S6B, Table S1). To narrow down this number, only SNARE proteins were chosen. Using this strategy, 10 SNARE proteins were obtained (Table 1). Of the 10 SNARE proteins, FocSso1, FocSec9, FocSso2 and FocSnc1 were the most abundant (Table 1). In yeast, Sso1 and Sso2 have high sequence similarity (74.24%) and probably arose from gene duplication events (Aalto et al., 1993). We found that FocSso1 and FocSso2 share only 31.45% sequence similarity. Phylogenetic and structural analyses revealed that the Sso2, Snc1 and Sec9 orthologues are very conserved in eukaryotes (Figure S7).

**TABLE 1:** Putative SNARE protein components that interact with GFP‐FocSso1.

Protein (yeast)	Accession (Foc)	Description	Coverage (%)	Unique peptides
Sso1	FOIG_01471	Syntaxin 1B/2/3	62	21
Sec9	FOIG_00476	Synaptosomal‐associated protein	50	15
Sso2	FOIG_07005	t‐SNARE coiled‐coil homology domain‐containing protein	31	6
Snc1	FOIG_02364	Vesicle‐associated membrane protein 4	30	5
Vti1	FOIG_07724	Vesicle transport through interaction with t‐SNAREs 1	22	3
Bos1	FOIG_10791	Protein transport protein BOS1	12	1
Bet1	FOIG_00379	Blocked early in transport 1	8	1
Gos1	FOIG_00867	Golgi SNAP receptor complex member 1	6	1
Sed5	FOIG_11941	Syntaxin 5	5	1
Nyv1	FOIG_04847	Synaptobrevin homologue YKT6	5	1

**FIGURE 3:**
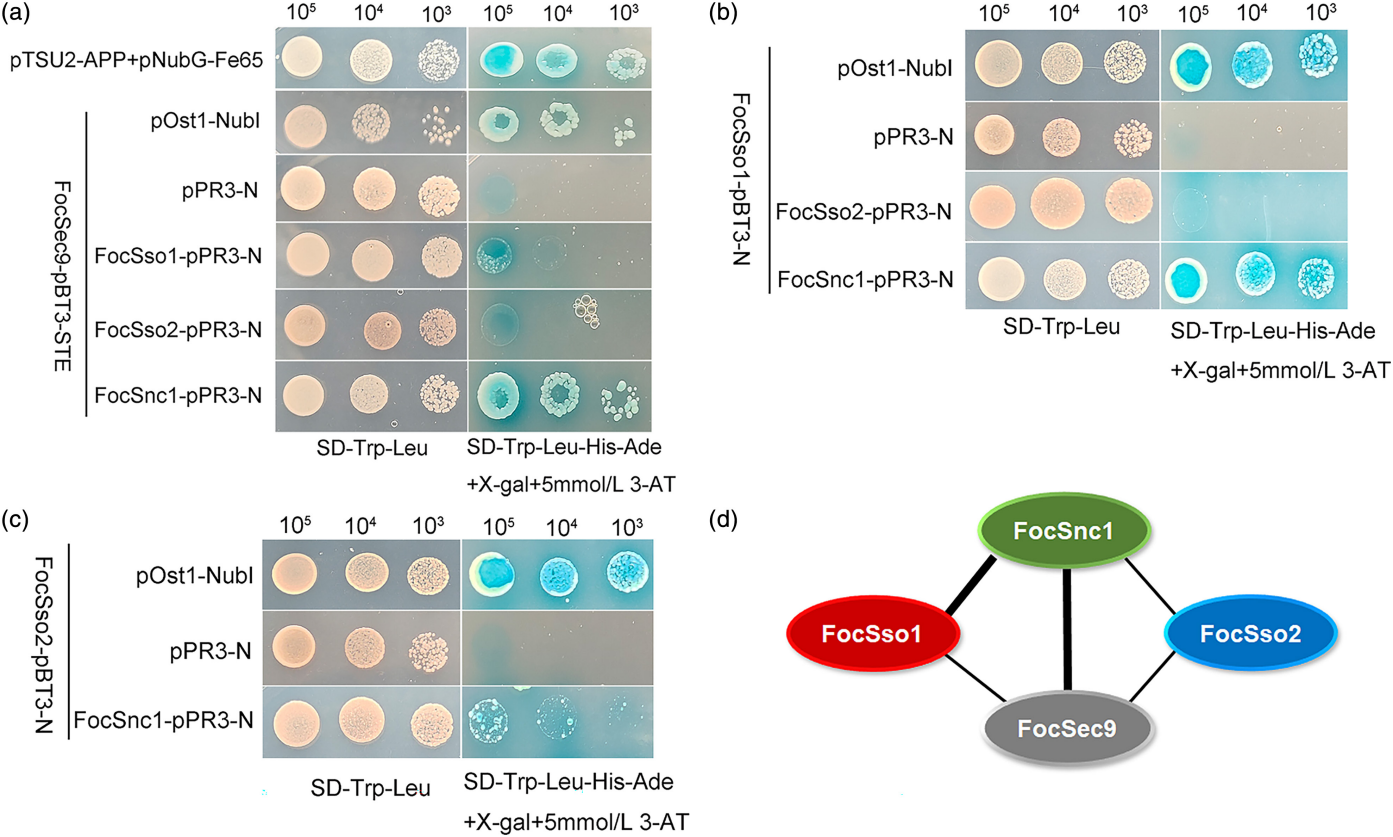
Yeast two‐hybrid (DUALhunter) assays showing possible interactions among the subunits of the SNARE complex. (a) Yeast transformants expressing FocSec9 as a bait and FocSso1, FocSso2 and FocSnc1 as prey. Constructs were grown on SD−Trp−Leu and SD−Trp−Leu−His−Ade to assay for α‐galactosidase (LacZ) activity. Interaction of pTSU22‐APP with pNubG‐Fe65 was used as positive control, that of FocSec9‐pBT3‐STE with pOst1‐Nub1 was used as functional control while that of FocSec9‐pBT3‐STE with pPR3‐N was used as negative control. All transformants were assayed at specified concentrations of 10^5^, 10^4^ and 10^3^ cells per μL. (b) Testing the interactions of FocSso1 with FocSso2 and FocSnc1. (c) Verification of interaction between FocSso2 and FocSnc1. (d) A model depicting physical interaction among the various SNARE complex subunits. The thick lines represent strong interaction (based on the above concentrations) between any two proteins while thin lines represent weak interaction between any two proteins.

**FIGURE 4:**
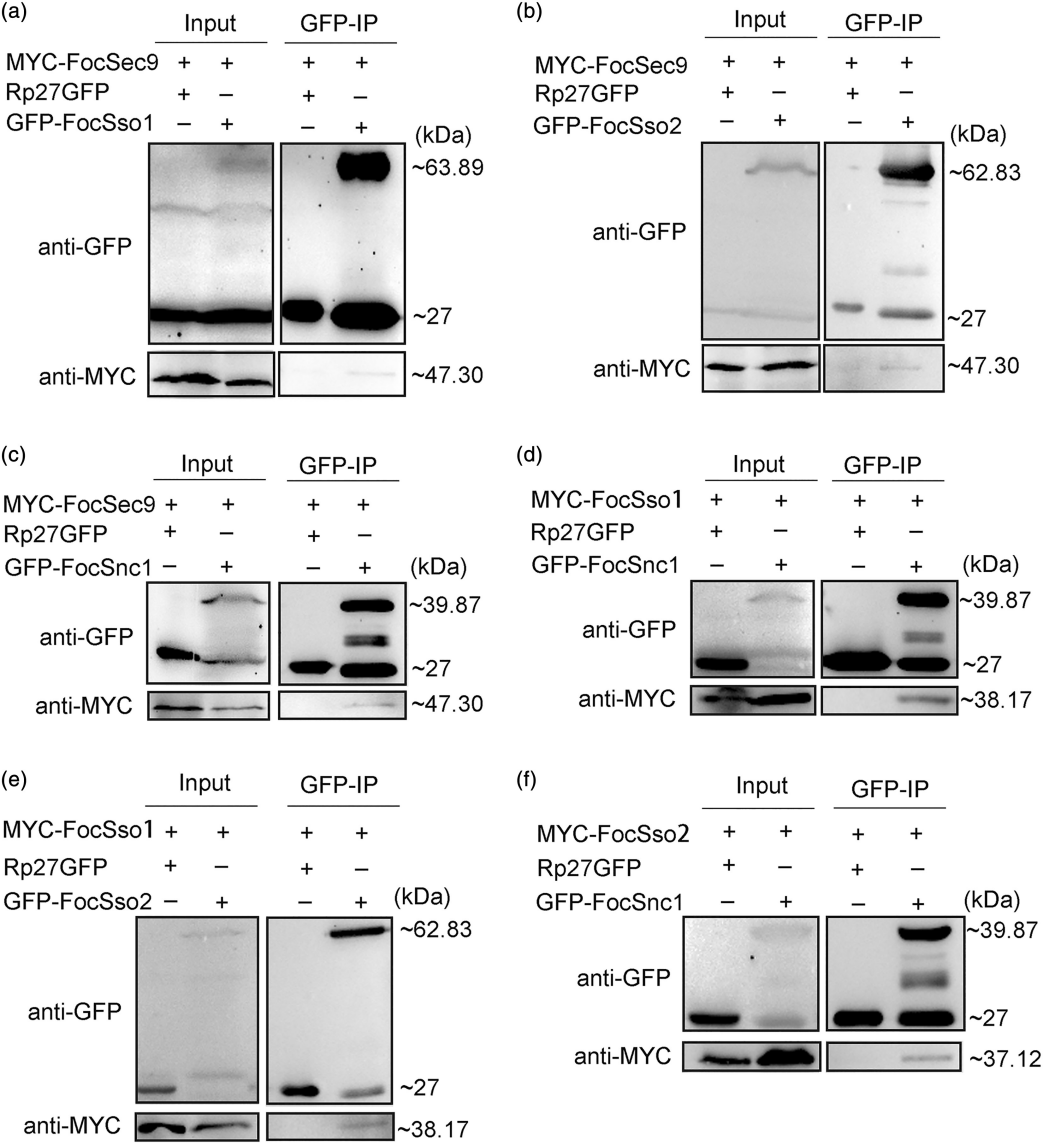
Co‐immunoprecipitation (Co‐IP) analysis showing how FocSso1–FocSso2–FocSnc1–FocSec9 form a SNARE complex. (a) Strains co‐expressing MYC‐FocSec9 and GFP‐FocSso1 proteins were immunoprecipitated with GFP trap beads. The immunoprecipitation (IP) signal (GFP‐FocSso1) and the Co‐IP signal (myc‐FocSec9) were detected by immunoblots probed with antibodies against GFP or MYC, respectively. The strains co‐expressing MYC‐FocSec9 and Rp27GFP (cytosolic GFP) proteins were used as negative controls. (b) Interaction of MYC‐FocSec9 with GFP‐FocSso2. (c) Interaction of MYC‐FocSec9 with GFP‐FocSnc1. (d) Interaction of MYC‐FocSso1 with GFP‐FocSnc1. (e) Interaction of MYC‐FocSso1 with GFP‐FocSso2. (f) Interaction of MYC‐FocSso2 with GFP‐FocSnc1.

We performed yeast two‐hybrid (Y2H) experiments to further verify the interactions among FocSso1, FocSso2, FocSnc1 and FocSec9 SNAREs. Considering that most SNAREs are membrane‐localized proteins and play a function in membrane fusion, we chose the DUAL membrane starter kits to test the interactions. We found that FocSec9 strongly interacted with FocSnc1 with high β‐galactosidase activity, but the interactions between FocSec9 and FocSso1, as well as between FocSec9 and FocSso2 were relatively weak (Figure 3a). Also, there was a strong interaction between FocSso1 and FocSnc1, but FocSso1 could not interact with FocSso2 (Figure 3b). Moreover, FocSso2 positively interacted with FocSnc1, but the affinity was low (Figure 3c). Based on these results, we proposed a physical interaction model for FocSso1/FocSso2–FocSnc1–FocSec9 SNARE complex in FocTR4 (Figure 3d). We further validated these results by co‐immunoprecipitation (Co‐IP) assays and the findings were in agreement with the Y2H data (Figure 4). Taken together, these results imply that both FocSso1 and FocSso2 independently form trimeric SNARE complexes with FocSnc1 and FocSec9.

### 2.6 FocSso2 and FocSnc1 are both important for FocTR4 virulence but play distinct roles in the fungal growth

Next, we characterized the biological roles of FocSso2, FocSnc1, and FocSec9 by a targeted gene replacement strategy (Figure S8). The *FocSSO2* and *FocSNC1* gene deletion mutants were identified by PCR and confirmed by Southern blots (Figure S8). However, we were unable to obtain deletion mutants for *FocSEC9* from more than 500 ectopic transformants after many attempts, suggesting that deletion of *FocSEC9* may be lethal to FocTR4, consistent with a previous report that deletion of yeast *SEC9* leads to cell death (Brennwald et al., 1994). Unlike FocSso1, phenotypic characterization showed that FocSso2 was dispensable for the fungal vegetative growth, but was required for conidiation (Figure S9). In addition, the growth of the Δ*Focsso2* mutant was affected by supplementation of stress‐inducing agents in a similar way to the wild type and the complemented strain (Figure S10). On the other hand, deletion of *FocSNC1* significantly perturbed the fungal growth and conidiation (Figure S9). Interestingly, we found that the Δ*Focsnc1* mutant showed much increased tolerance to the hyperosmotic stress‐inducing agents NaCl and KCl compared to the wild‐type strain (Figure S11). These data indicate that FocSso2 and FocSnc1 have distinct functions in FocTR4 with respect to growth and responses to environmental stress.

**FIGURE 5:**
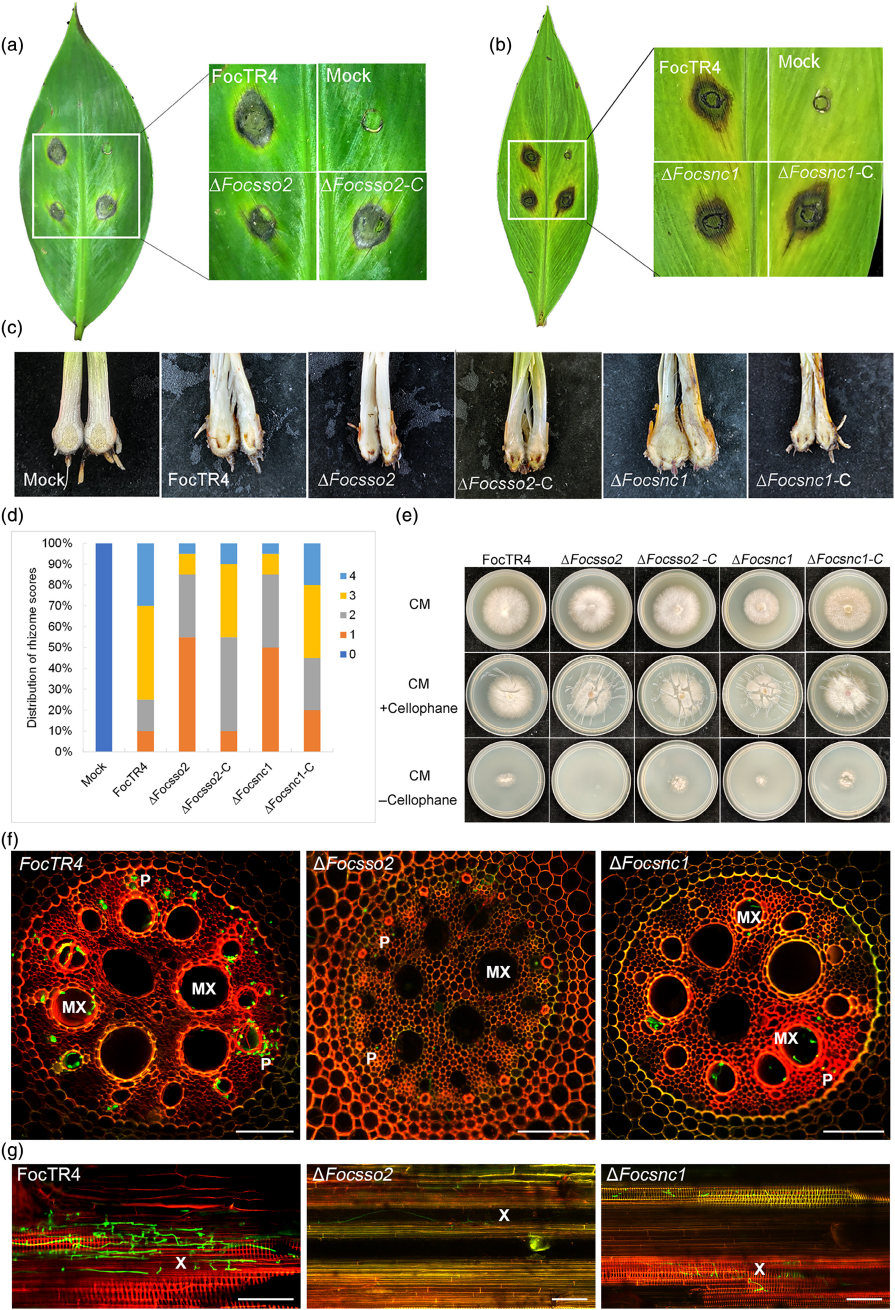
Effects of *FocSSO2* and *FocSNC1* deletions on virulence of *Fusarium oxysporum* f. sp. *cubense* tropical race 4 (FocTR4). (a) Pathogenicity assay of ∆*Focsso2* mutant on banana leaves. (b) Pathogenicity assay of ∆*Focsnc1* mutant on banana leaves. (c) Degree of damage inflicted by ∆*Focsso1* and ∆*Focsso2* mutants on banana roots. (d) Disease indexes based on the degree of damage inflicted by the indicated strains on banana roots. (e) Cellophane penetration ability of the indicated strains. ∆*Focsso2* was unable to penetrate a single layer of the cellophane after 2 days of incubation, while ∆*Focsnc1* could effectively penetrate cellophane to establish a relatively small colony. CM, complete medium. (f, g) Cross and longitudinal sections of banana roots, respectively, infected with the indicated strains harbouring ectopically expressed green fluorescent protein (GFP). The images were taken at 3 days post‐inoculation; abundant invasive hyphae could be observed in the root tissues infected with GFP‐tagged FocTR4, but only a few of such signals were observed in those infected with ∆*Focsso2* and ∆*Focsnc1*. MX, metaxylem; P, phloem; X, xylem. Bars, 100 μm.

To investigate whether *FocSSO2* and *FocSNC1* are important for the fungal virulence, we inoculated the mutants of each gene on banana leaves and roots. Like the Δ*Focsso1* mutant, both Δ*Focsso2* and Δ*Focsnc1* mutants had significantly reduced pathogenicity on banana seedlings compared to the controls (Figure 5a–d). In vitro and in vivo penetration assays showed that loss of *FocSSO2* and *FocSNC1* impaired FocTR4 penetration and colonization (Figure 5e–g). Taken together, these results indicate that FocSso2 and FocSnc1 play distinct roles in the fungal growth, but both proteins are important for the fungal virulence.

### 2.7 FocSso2 is only present in the PM of old cells, while FocSnc1 is widely distributed in the PM, vesicles and hyphal apex

**FIGURE 6:**
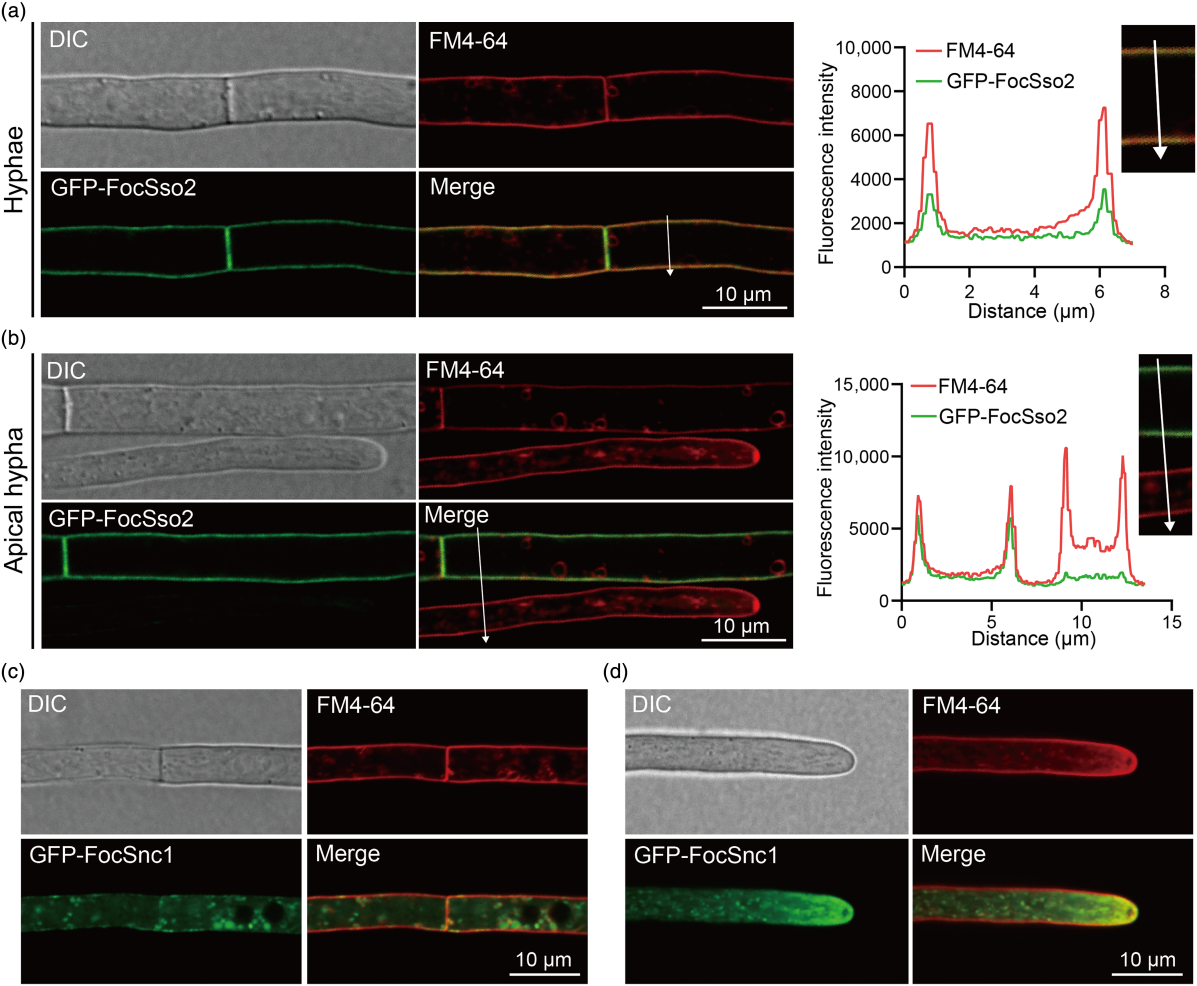
Subcellular localizations of FocSso2 and FocSnc1. FocSso2 only localizes to the plasma membrane (PM) while FocSnc1 localizes to both the PM and transport vesicles and also accumulates at growing hyphal tips. (a) Hyphae expressing GFP‐FocSso2 were stained with FM4‐64 and observed under a confocal microscope. The GFP‐FocSso2 is localized to the PM and septa at which it also co‐localized with the endocytic dye FM4‐64. A line scan graph was generated at the position indicated by an arrow to show the relative co‐localization of GFP‐FocSso2 (green) and FM4‐64 (red). (b) GFP‐FocSso2 fluorescence can only be found in old hyphae; the fluorescence signal is completely absent in apical hyphae. FM4‐64 was used in this instance to visualize hyphae and compare the fluorescence intensity with GFP‐FocSso2. (c) GFP‐FocSnc1 is localized to the PM and transport vesicles in the fungal hyphae. (d) GFP‐FocSnc1 also accumulates at the hyphal apex. DIC, differential interference contrast; GFP, green fluorescent protein.

To dissect the functional mechanisms of FocSso2 and FocSnc1 in Foc, we transformed *GFP‐FocSSO2* and *GFP‐FocSNC1* fusion constructs into the Δ*Focsso2* and Δ*Focsnc1* mutants, respectively. Phenotypic analyses revealed that the transformants were restored in all the defects observed in each mutant (Figure 5, Figures [Link], [Link], [Link]). Next, we used a laser confocal microscope to observe the subcellular localizations of GFP‐FocSso2 and GFP‐FocSnc1. We found that the GFP‐FocSso2 only localized to the PM in old hyphal cells, displaying bright and uniform fluorescence signals (Figure 6a,b). However, the GFP‐FocSso2 signal appeared very weak at the apex of growing hyphae (Figure 6b). In contrast, GFP‐FocSnc1 displayed various localization patterns in both old and young hyphal cells, including the PM, punctate vesicles and hyphal apexes (Figure 6c,d). FRAP and time‐lapse imaging experiments further confirmed that FocSnc1 was involved in the continuous movement of vesicles and polarized protein secretion (Figure S12, Video S3). Overall, these results reveal that FocSso2 and FocSnc1 have distinct spatial localizations in FocTR4. Specifically, FocSnc1 is involved in vesicle trafficking and exocytosis at the hyphal apex.

### 2.8 FocSso1 and FocSnc1 are functionally interdependent

**FIGURE 7:**
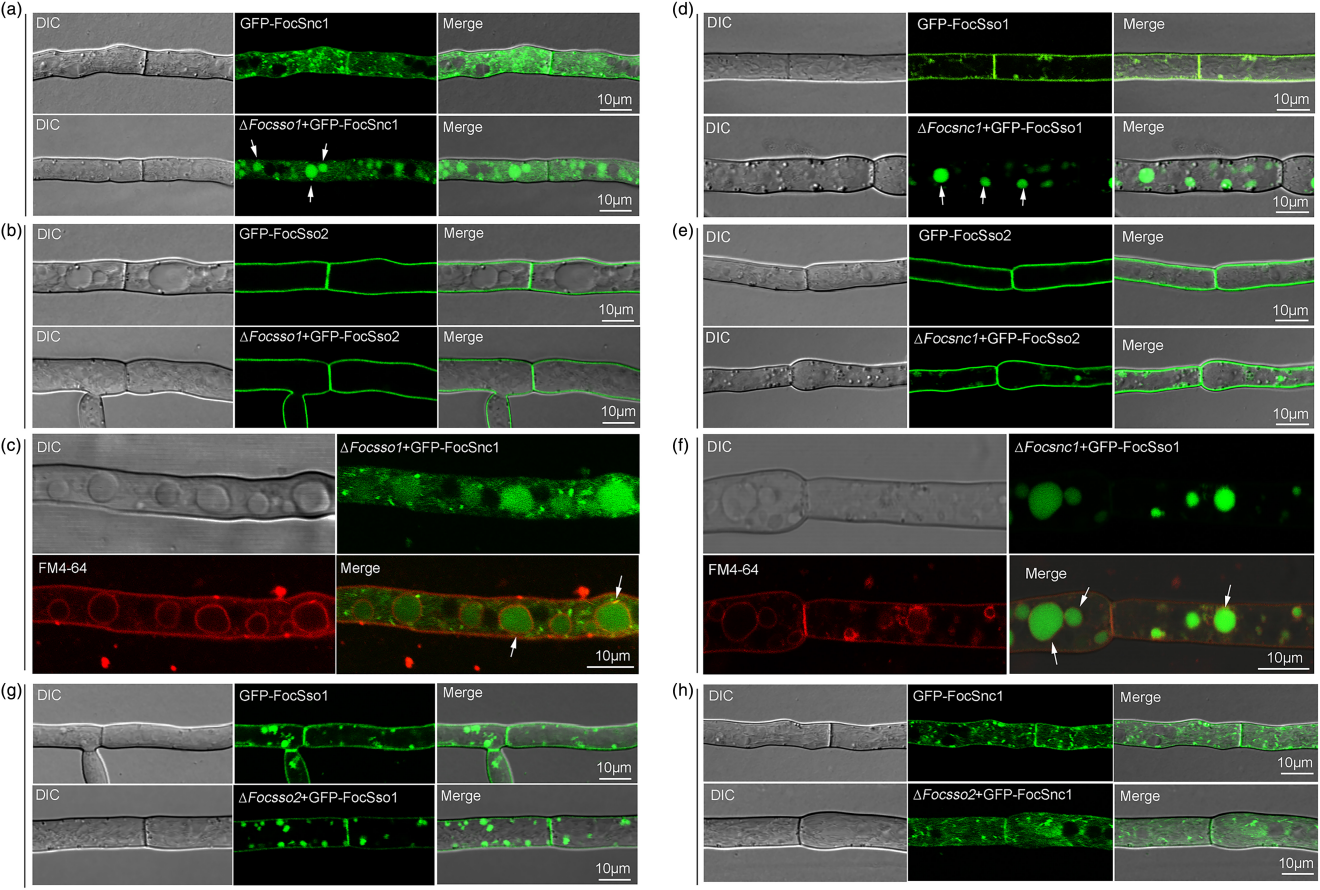
Functional relationships of FocSso1, FocSso2 and FocSnc1 proteins. (a) Deletion of *FocSSO1* causes GFP‐FocSnc1 to be mislocalized into some vacuolar structures. (b) Deletion of *FocSSO1* does not affect the localization of GFP‐FocSso2. (c) GFP‐FocSnc1 is mislocalized into the vacuoles in Δ*Focsso1* mutant. FM4‐64 was used for staining of vacuole membrane. (d) Deletion of *FocSNC1* causes GFP‐FocSso1 to be mislocalized into some vacuolar structures. (e) Deletion of *FocSNC1* does not affect the localization of GFP‐FocSso2. (f) GFP‐FocSso1 is mislocalized into the vacuoles in Δ*Focsnc1* mutant. FM4‐64 was used for staining of vacuole membrane. (g) Deletion of *FocSSO2* does not affect the localization of GFP‐FocSso1. (h) Deletion of *FocSSO2* does not affect the localization of GFP‐FocSnc1. Arrows indicate vacuoles; DIC, differential interference contrast; GFP, green fluorescent protein.

Although FocSso1, FocSso2 and FocSnc1 are all associated with the PM, their localizations are not completely the same and they have distinct biological functions. To explore the roles of FocSso1 in maintaining the stability of the SNARE complex, we investigated the subcellular localizations of FocSso2 and FocSnc1 in the Δ*Focsso1* mutant by laser confocal microscopy. The localization of GFP‐FocSnc1 was significantly altered in the mutant (Figure 7a), while deletion of *FocSSO1* did not influence the PM localization of GFP‐FocSso2 (Figure 7b). In the Δ*Focsso1* mutant, GFP‐FocSnc1 showed a predominantly aggregate distribution in some vacuole‐like structures and failed to localize to the PM in hyphae (Figure 7a). To confirm that GFP‐FocSnc1 is mislocalized into the degradative vacuoles, we used the endocytic dye FM4‐64 to visualize vacuolar membranes. FM4‐64 staining demonstrated that GFP‐FocSnc1 was mis‐sorted into the vacuole degradation pathway in the absence of *FocSSO1* (Figure 7c). To understand whether the other components of the SNARE complex have a similar function to FocSso1 in maintaining the stability of the complex, we analysed the localizations of FocSso1 and FocSso2 in the Δ*Focsnc1* mutant. The results showed that FocSnc1 was dispensable for normal localization of FocSso2, but was important for the correct localization of FocSso1 (Figure 7d–f). However, deletion of *FocSSO2* did not affect the normal localizations of FocSso1 and FocSnc1 (Figure 7g,h). Taken together, these results indicate that FocSso1 and FocSnc1 are functionally interdependent and are essential for SNARE complex stability.

## 3 DISCUSSION

**FIGURE 8:**
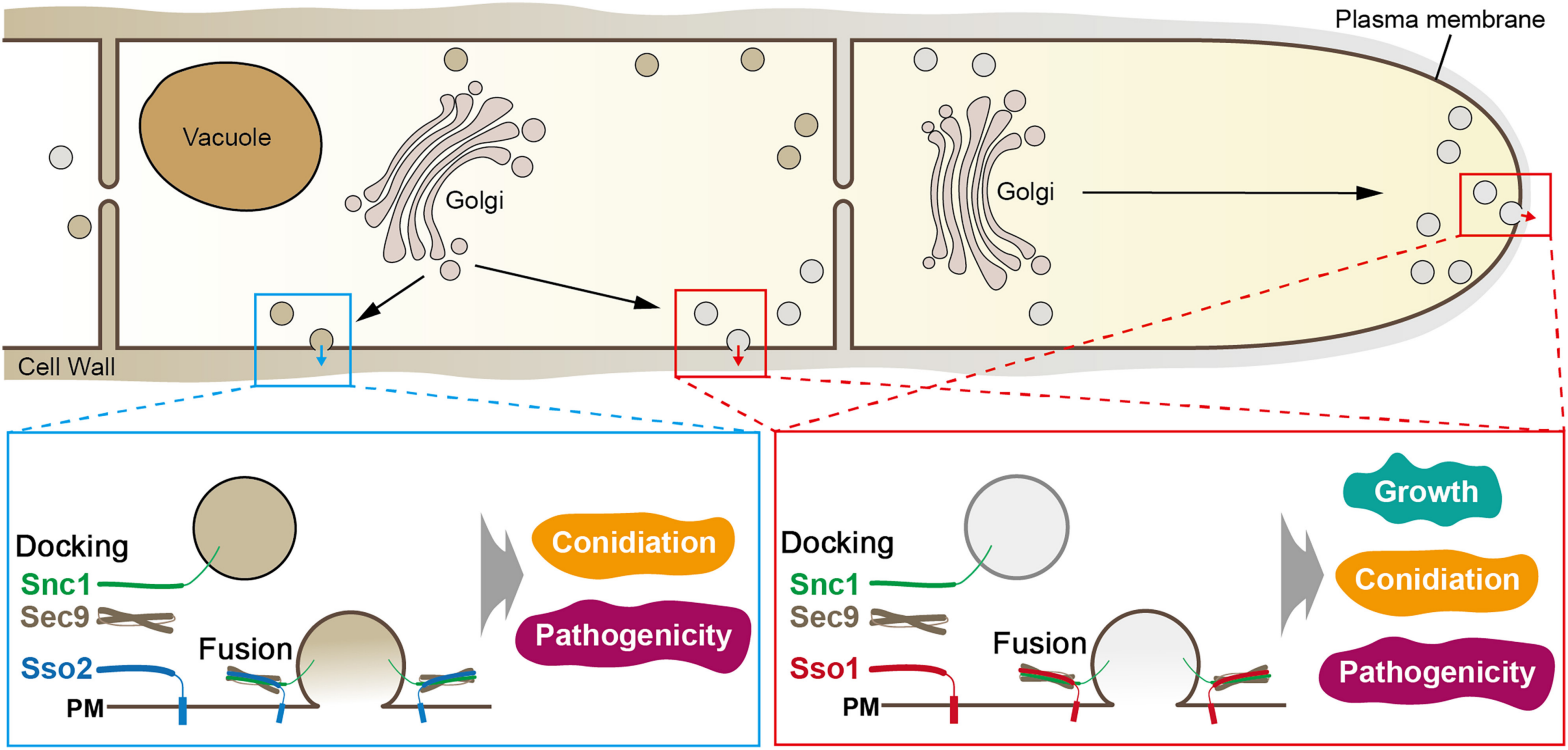
A working model for the mechanistic functions of the components of plasma membrane (PM) SNARE complex in relation to growth, conidiation and pathogenicity of the banana Fusarium wilt fungus. Secretory vesicles dock and fuse with the PM in a constitutive fashion via formation of two sets of PM‐associated SNARE complex. The first complex consists of Sso1, Sec9 and Snc1, which are localized in young hyphal apex and all hyphal PM. The Sso1–Sec9–Snc1 complex‐mediated exocytosis regulates hyphal growth, conidiation and pathogenicity. The second complex consists of Sso2, Sec9 and Snc1, which are localized in old hyphal PM. The Sso2–Sec9–Snc1 complex‐mediated exocytosis regulates conidiation and pathogenicity. The distinct locations of the two sets of SNARE complex could also serve as sites for exocytosis of different extracellular enzymes.

In eukaryotic cells, SNARE proteins are well conserved and form a complex that leads to membrane fusion between vesicles, organelles, and PMs (Jahn & Scheller, 2006). In this study, we characterized two PM‐associated SNARE complexes in the banana Fusarium wilt fungus FocTR4 (Figure 8). Our live‐cell imaging techniques support that FocSso1 is involved in the docking and fusion of exocytic vesicles with the PM, and deletion of *FocSSO1* affects protein secretion as well as the fungal ability to penetrate its host. We also identified the components and forms of PM‐associated SNARE complexes in FocTR4. Specifically, two distinct complexes FocSso1–FocSec9–FocSnc1 and FocSso2–FocSec9–FocSnc1 were established, and both coordinately facilitate docking and fusion of exocytic vesicles with the PM in a spatially and functionally specific manner (Figure 8). Disruption of any component of these SNARE complexes affects the FocTR4 invasion and colonization of the host. In addition, the regulatory factors for the stability of the PM SNARE complexes have been revealed. This comprehensive study sheds light on how the spatial assembly of a functional SNAREpin is achieved and establishes the link between exocytic vesicles trafficking to the PM, and growth and pathogenesis of FocTR4.

*Sso1* and *Sso2* genes were first characterized in *S. cerevisiae* and have been identified to mediate vesicular transport (Aalto et al., 1993). Subsequently, studies showed that Sso1 is important for spore formation, whereas Sso1 and Sso2 have redundant functions with respect to vegetative growth (Jäntti et al., 2002; Nakanishi et al., 2006). In the model filamentous fungus *N. crassa*, nsyn1 (Sso1 homologue) was shown to be important for hyphal development, conidiation and male fertility, while nsyn2 (Sso2 homologue) is important for hyphal branching and ascospore development (Gupta et al., 2003). Interestingly, the Sso1 homologues in plant pathogens (like the wheat scab pathogen *F. graminearum*, maize stalk rot pathogen *F. verticillioides*, Verticillium wilt pathogen *V. dahliae* and rice blast pathogen *M. oryzae*) are important for the fungal virulence (Giraldo et al., 2013; Hong et al., 2010; Wang et al., 2018; Zhang et al., 2019). However, the role of Sso1 in the pathogenicity of phytopathogens remains unclear. Our study showed that loss of *FocSSO1* in the FocTR4 leads to significant reduction in the virulence of the fungus on banana leaves and corms. In addition, we confirmed that the observed defect in pathogenesis is largely due to failure of the mutant to penetrate it host and eventually influence colonization and spreading inside the host. Our data support that FocSso1 is involved in protein secretion because the abundance of secreted proteins was significantly reduced in the Δ*Focsso1* mutant. The main challenge for future studies is to identify and characterize the proteins whose secretions are mediated by the FocSso1 protein. Achieving this will further our understanding of the mechanism of FocSso1 function in fungal pathogenesis.

SNAREs assemble into helices in a stoichiometric fashion to form a SNARE complex, the formation of which places vesicles and target membranes in very close apposition, a prerequisite for fusion (Sutton et al., 1998). While members of the SNARE family are largely conserved across fungi (Gupta & Brent Heath, 2002; Kienle et al., 2009), SNARE protein interactions and assembly of a functional SNAREpin have not been characterized in filamentous fungi. Here, we used a high‐resolution interactome analysis of GFP‐trap‐precipitated GFP‐FocSso1 to screen its interacting partners in FocTR4. This screening identified a total of 705 FocSso1‐interacting proteins, including 10 SNARE proteins. In addition to the FocSso1 itself, three SNARE proteins, FocSec9, FocSso2 and FocSnc1, were identified as FocSso1 high‐confidence precipitates. Subsequently, we employed in vivo strategies, such as Co‐IP and Y2H, to determine the interactions and combinations of these SNARE proteins to form a complex. Our results showed that FocSso1 does not physically interact with FocSso2, while others showed positive interactions in different combinations. Therefore, we proposed that the FocSso1 constitutes a ternary SNARE complex with FocSnc1 and FocSec9. Similarly, FocSso2 constitutes a ternary SANRE complex with FocSnc1 and FocSec9. To our knowledge, this is the first study that uncover the interaction patterns of PM‐associated SNARE proteins in filamentous fungi.

Although our data show that FocSso2 is also required for fungal penetration and spread in banana seedling similar to FocSso1, the two proteins do have obvious functional differences. Firstly, the Δ*Focsso1* mutant was reduced by 70% in hyphal growth compared to the wild‐type strain, while Δ*Focsso2* mutant had a similar hyphal growth rate to the wild type. Consistently, orthologues from *G. zeae*, GzSYN1 and GzSYN2, also regulate distinct growth phenotypes. Functional analysis of these two genes showed that *GzSYN1* is important for the fungal growth while *GzSYN2* is dispensable for growth (Hong et al., 2010). In addition, we also found that the Δ*Focsso1* mutant had increased tolerance to membrane stress induced by SDS, CR and CFW, while Δ*Focsso2* had similar sensitivity to the wild type. These data indicate that the FocSso1 and FocSso2 proteins have diverse and distinct roles in FocTR4, despite having certain sequence similarity. Secondly, the subcellular localizations of GFP‐FocSso1 and GFP‐FocSso2 are not completely the same. The GFP‐FocSso1 localizes to the PM, hyphal apexes and mobile vesicles in hyphae. Time‐lapse confocal imaging showed that GFP‐FocSso1‐labelled vesicles in the fungal hyphae are able to move towards and/or transiently fused with the PM. However, we could not detect the fluorescence signal of GFP‐FocSso2 at the tips of growing hyphae; the GFP‐FocSso2 only showed steady‐state localization at the PM of old hyphae. In *Trichoderma reesei*, the two SNARE proteins Sso1 and Sso2 were found to be involved in different secretory processes (Valkonen et al., 2007). While Sso2 was found in the apical compartments of actively growing hyphae, Sso1 was detected in older, non‐growing hyphae (Valkonen et al., 2007). Our results are therefore consistent with the findings in *T. reesei*, while the naming convention of Sso1 and Sso2 was reversed in *T. reesei*. In *M. oryzae*, the Sso1 orthologue localizes in invasive hyphae as a vesicle‐like structure near the biotrophic interfacial complex in invaded rice cells (Giraldo et al., 2013), while Sso2 orthologue only localizes to PM, displaying uniform fluorescence (Chen et al., 2023). The vesicular localization patterns of Sso1 in different organisms together imply that the protein mediates docking and fusion of vesicles with the PM. This observation also suggests that FocSso1 plays a dominant role in exocytosis because it is widely distributed in the cells. Thirdly, our Y2H data showed that FocSso1 and FocSso2 do not interact physically. Furthermore, FocSso1 and FocSso2 are not functionally interdependent, because loss of FocSso1 does not affect the localization of FocSso2, and vice versa. Taken together, these results imply that Sso1 and Sso2 are not functionally redundant in filamentous fungi, and they form two distinct SNARE complexes that mediate the fusion of different secretory vesicles in filamentous fungi. Additionally, this finding may suggest new genetic specialization through evolution.

After the assembly of SNARE proteins into a complex, the factors that maintain the stability of such complex are still unclear. To explore the roles of each component of the FocSso1/FocSso2–FocSnc1–FocSec9 SNARE complex in the stability of the complex, we analysed possible changes in the subcellular localizations of the different components in individual mutants of such components. Our data showed that FocSso1 and FocSnc1 are functionally interdependent and are essential for the SNARE complex stability. It is not surprising that FocSso1 and FocSnc1 regulate the formation and functions of PM‐associated SNARE complexes considering the complicated localization patterns of the proteins and the severe effects of deleting any one of them. Future work on structural biology with different mutations on this PM SNARE complex may offer a more detailed working mechanism.

## 4 EXPERIMENTAL PROCEDURES

### 4.1 Strains and culture conditions

*Fusarium odoratissimum* strain 58 was employed for gene knockout and transformation of fluorescent strains (Yun et al., 2019; Table S2). All strains were cultured at a temperature of 28°C on both CM and MM for growth analysis (Table S2). The cultures were then preserved in 20% glycerol at a temperature of −80°C in the form of mycelial suspensions. Additionally, the strains were cultured on both potato dextrose broth (PDB) and Spezieller Nährstoffarmer agar (SNA) for conidiation analysis. The banana seedlings used in the study belonged to the Cavendish banana (AAA cultivar).

### 4.2 Gene deletions and complementations

All the mutant strains used were obtained by protoplast transformation of the wild‐type strains using the product of double‐joint PCR (Yu et al., 2004). The primers used for amplifying both upstream and downstream segments of all genes are detailed in Table S3. Hygromycin‐resistant transformants were screened using PCR with two pairs of primers (Table S3). Positive transformants were further verified by Southern blot using Digoxigenin High Prime DNA Labelling and Detection Starter Kit I (Roche). To produce fluorescent vectors, the native promoter regions of the target genes, the GFP fragment, and the gene ORFs were PCR‐amplified, followed by cloning into pKNT vector using a ClonExpress MultiS One Step Cloning Kit (Vazyme Biotech). Details of the plasmid construction are listed in Table S4. Subsequently, these fluorescent vectors were introduced into the mutant strain to generate complemented strains.

### 4.3 Growth and conidiation assays

To perform growth analysis, mycelial blocks were excised from the colony edges of 3‐day‐old CM cultures of the fungal strains and inoculated on both CM and MM in 7 cm Petri dishes. The plates were subsequently incubated at 28°C for 3 days. We measured the colony diameters and observed any alterations in the colony morphology, which were photographed. For microconidiation analysis, the mycelial blocks were transferred to PDB and incubated at 28°C with constant agitation at 180 rpm, for 3 days. After incubation, the mycelia developed were filtered out, and microconidia were counted using a haemocytometer. For macroconidiation analysis, the mycelial blocks were inoculated on 7 cm SNA plates. The plates were then incubated at 28°C for 3 days and the macroconidia were rinsed with double‐distilled water and counted using a haemocytometer. These experiments were repeated three times independently.

### 4.4 Pathogenicity analysis

For pathogenicity assay, mycelial blocks from each strain were inoculated on the leaves of young banana seedlings. The leaf tissues in direct contact the fungal mycelia were subjected to same degree of tissue contusion. The inoculated seedlings were incubated at 28°C with humidity maintained for 3 days. Also, roots of some banana seedlings grown in soil were rinsed with sterile water and the root tips were subsequently removed. The roots were then immersed in a conidial suspension at a concentration of 10^6^ conidia/mL for 8 h. Afterwards, they were transplanted into the soil and allowed to grow for 6 weeks. The root bulbs were subsequently isolated for assessment of root pathogenicity. The pathogenicity scoring system was adopted from Widinugraheni et al. (2018). Three biological replicates were involved.

### 4.5 Penetration and root infection analysis

The fungal strains were cultured on CM for 3 days. Mycelial discs, about 6 mm in diameter, were taken from the edge of the colony and inoculated on fresh CM covered with a layer of cellophane membrane. After 3 days of incubation at 28°C, the cellophane was removed, and the fungal growth on the medium was observed after another 2 days of incubation at 28°C. As per the method described by Li et al. (2017), root infection experiments were conducted. At 3 dpi, root sections located 0.5 cm from the inoculation site were examined by confocal microscopy to assess the infection. All fungal strains were labelled with GFP, and the banana root cells were observed using a 561 nm laser.

### 4.6 Y2H assay

To verify the interactions between FocSso1, FocsSso2, FocSec9 and FocSnc1, their cDNA sequences were individually fused to bait plasmids pBT3‐N and pBT3‐STE, as well as prey plasmid pPR3‐N (Dualsystems Biotech), based on the DUALmembrane starter kits User Manual (Dualsystems Biotech). These constructs were co‐transformed into NMY51 cells. Subsequently, the transformed cells were cultured on SD−Trp−Leu and SD−Trp−Leu−His−Ade + X‐Gal agar plates at 30°C for 3–5 days. After selecting colonies, they were suspended in distilled water at different concentrations and spotted onto SD−Trp−Leu and SD−Trp−Leu−His−Ade + X‐Gal + 5 mM 3‐AT agar plates. The plates were then incubated at 30°C for 4 days, and interactions were observed.

### 4.7 GFP pull‐down and Co‐IP assays

The GFP‐ and GFP‐FocSso1‐expressing strains were cultured in liquid CM with constant agitation at 28°C for 2 days. The resulting mycelia were filtered, washed with sterile distilled water and ground into powder in liguid nitrogen. The powder was suspended in lysis buffer containing a cocktail of protease inhibitor for 30 min. The suspension was then centrifuged and the supernatant was collected. About 30 μL of GFP‐Trap_A beads (ChromoTek Inc., Planegg‐Martinsried) was added to the supernatant, and the mixture was incubated for 4 h at 4°C. The proteins bound to the beads were then eluted by heating at 100°C for 10 min. Subsequently, mass spectrometry analysis was performed following a previously established method (Zheng et al., 2018). The protein samples for Co‐IP were extracted using the same method as described previously. Following boiling, the protein samples were separated using 10% SDS‐PAGE and subsequently analysed using western blotting. The analysis involved the use of anti‐GFP antibody (GFPTag (7G9) mouse mAb; Abmart), goat anti‐mouse IgG horseradish peroxidase (HRP) (Abmart), and anti‐Myc antibody (HRP anti‐Myc tag antibody; Abcam).

### 4.8 Extraction and quantification of secreted proteins

Secreted proteins were extracted following the method described by Zheng et al. (2022). The secreted protein concentration was quantified using a Bradford protein assay kit (Beyotime).

### 4.9 Confocal microscopy

Live cell fluorescent images were captured using a spinning‐disk confocal microscope (Nikon). The microscope was equipped with a Yokogawa CSU‐W1 spinning‐disk confocal system and a 100×/1.45 NA oil objective lens was used. The images were captured using a 16‐bit digital ORCA‐Fusion BT CoaXpress set (Hamamatsu Photonics KK). FRAP experiments were conducted using a laser set (405 nm high power laser) at an intensity of 10% with a photobleaching time of 50 μs. Images were captured both before and after photobleaching using the confocal microscope.

## Supporting information


**Figure S1.** Phylogenetic analysis of Sso1 orthologues in eukaryotes. (A) A neighbour‐joining tree was constructed based on the amino acid sequences of the Sso1 orthologues. Numbers at the nodes represent the percentage of occurrence in 10,000 bootstrap replicates. (B) Domain structure of Sso1 orthologues in eukaryotes. The conserved syntaxin domains (blue) are shown. (C) Prediction of transmembrane helices in FocSso1 proteins.

**Figure S2.** Gene deletion strategy and Southern blot assays. (A) Targeted gene‐replacement strategy for *FocSSO1* is shown. The primer pairs F1 and R1, F3 and R3 were used to generate the gene replacement constructs. Primers F2 and R2 were used for mutant screening and identification. (B) Southern blot analysis for confirmation of gene deletions. NcoΙ‐digested genomic DNAs showed a 6.87 kb band in the wild type (WT) and a 3.81 kb band in the mutants.

**Figure S3.** Role of FocSso1 in the vegetative growth and sporulation of the Fusarium wilt fungus. (A) Colony morphology and growth of the wild type (FocTR4), ∆*Focsso1* and ∆*Focsso1‐C* on complete medium (CM) and minimal medium (MM). (B) Graphical representation of the colony diameters of the indicated strains. (C) Number of microconidia produced by the indicated strains in potato dextrose broth. (D) Number of macroconidia produced by the indicated strains in Spezieller Nährstoffarmer agar. Values are presented as means ± *SD* (standard deviation) calculated from three independent experiments. **p < 0.05, ***p < 0.001.

**Figure S4.** Continuous movement of GFP‐FocSso1 to hyphal apexes and septa. (A) Representative time series images of GFP‐FocSso1 at the hyphal apexes immediately after photobleach (*t* = 0 s). Fluorescence recovery after photobleaching (FRAP) at the bleaching site after 3 min. (B) Representative time series images of GFP‐FocSso1 in the septa immediately after photobleach (*t* = 0 s). Fluorescence recovery after photobleaching (FRAP) at the bleaching site after 6 min. Bar, 10 μm.

**Figure S5.** Sensitivity of the wild‐type strain (FocTR4), *FocSSO1* gene deletion mutant (Δ*Focsso1*) and complemented strain (Δ*Focsso1‐C*) to osmotic, oxidative and cell wall stresses. (A) Colonies of the indicated strains on complete medium (CM) supplemented with 0.02% (wt/vol) SDS, 0.7 M NaCl, 36 mM H_2_O_2_, 200 μg/mL Congo red (CR) and 200 μg/mL calcofluor white (CFW). (B) Mycelial radial growth inhibition rates were quantified 3 days after culturing the strains on CM with different stress‐inducing agents. **p < 0.05.

**Figure S6.** Identification of FocSso1 interacting proteins. (A) Number of identified proteins by affinity purification and mass spectrometry analysis in GFP‐FocSso1 and wild‐type (WT)‐GFP strains. (B) 705 proteins were specifically identified in GFP‐FocSso1 interactome but not in WT‐GFP.

**Figure S7.** Phylogenetic analysis of Snc1, Sso2 and Sec9 orthologues in eukaryotes. (A) Phylogenetic tree and domain architectures of Snc1 orthologues in eukaryotes. (B) Phylogenetic tree and domain architectures of Sso2 orthologues in eukaryotes. (C) Phylogenetic tree and domain architectures of Sec9 orthologues in eukaryotes.

**Figure S8.** Targeted gene replacement strategy and Southern blot assays for *FocSNC1* and *FocSSO2* gene deletions. (A) Targeted gene replacement strategy for *FocSNC1* is shown. NcoΙ‐digested genomic DNAs showed a 5.91 kb band in the wild type (WT) and a 1.88 kb band in the mutants. (B) The targeted gene‐replacement strategy for *FocSSO2* is shown. NdeΙ‐digested genomic DNAs showed a 3.54 kb band in the WT and a 1.62 kb band in the mutants.

**Figure S9.** Phenotypic characterization of ∆*Focsso2* and ∆*Focsnc1* mutants. (A) Colony morphology and growth of the indicated strains on complete medium (CM) and minimal medium (MM). (B) Bar graph representation of the colony diameters of the indicated strains. (C) Number of microconidia produced by the indicated strains following growth in potato dextrose broth. (D) Number of macroconidia produced by the indicated strains following growth in Spezieller Nährstoffarmer agar. Values are presented as means ± *SD* calculated from three independent experiments. **p < 0.05, ***p < 0.001.

**Figure S10.** Sensitivity of the wild‐type strain (FocTR4), *FocSSO2* gene deletion mutant (Δ*Focsso2*) and complemented strain (Δ*Focsso2‐C*) to osmotic, oxidative and cell wall stresses. (A) Colonies of the indicated strains on complete medium (CM) supplemented with 0.02% (wt/vol) SDS, 0.7 M NaCl, 36 mM H_2_O_2_, 200 μg/mL Congo red (CR) and 200 μg/mL calcofluor white (CFW). (B) Mycelial radial growth inhibition rates were quantified after culturing the strains on CM containing different stress‐inducing agents at 3 days post‐inoculation.

**Figure S11.** Sensitivity of the wild‐type strain (FocTR4), *FocSNC1* gene deletion mutant (Δ*Focsnc1*) and complemented strain (Δ*Focsnc1‐C*) to osmotic, oxidative and cell wall stresses. (A) Colonies of the indicated strains on complete medium (CM) supplemented with 0.02% (wt/vol) SDS, 0.7 M NaCl, 36 mM H_2_O_2_, 200 μg/mL Congo red (CR) and 200 μg/mL calcofluor white (CFW). (B) Mycelial radial growth inhibition rates were quantified after culturing the strains on CM containing different stress‐inducing agents at 3 days post‐inoculation. (C) Colonies of the indicated strains on CM supplemented with 0.7 M KCl and 0.7 M NaNO_3_. (D) Mycelial radial growth inhibition rates were quantified after culturing the strains on CM containing different stress‐inducing agents at 3 days post‐inoculation. **p < 0.05.

**Figure S12.** Continuous movement of GFP‐FocSnc1 to hyphal apex and plasma membrane. (A) Representative time series images of GFP‐FocSnc1 on the hyphal apex immediately after photobleach (*t* = 0 s). Fluorescence recovery after photobleaching (FRAP) recovery in the photobleaching site after 1 min. (B) Representative time series images of GFP‐FocSnc1 on the plasma membrane of immediately following the photobleach (*t* = 0 s). Fluorescence recovery after photobleaching (FRAP) at the bleaching site after 1 min. Bar, 10 μm.

**Table S1.** Putative proteins that interact with GFP‐FocSso1.

**Table S2.** Wild‐type and mutant strains of fungi used in this study.

**Table S3.** PCR primers used in this study.

**Table S4.** Plasmids used in this study.

**Video S1.** Dynamic tracking of vesicular transport of GFP‐FocSso1 to the plasma membrane.

**Video S2.** Dynamic tracking of vesicular transport of GFP‐FocSso1 to the hyphal tip in growing hypha.

**Video S3.** Dynamic tracking of vesicular transport of GFP‐FocSnc1 to the hyphal tip in growing hypha.

## Data Availability

The data needed for evaluation of the conclusions in the paper are presented in the paper and/or the Supporting Information.
