# Supplementary material for: Two distinct SNARE complexes mediate vesicle fusion with the plasma membrane to ensure effective development and pathogenesis of Fusarium oxysporum f. sp. cubense
Source: Mol Plant Pathol. 2024 Mar 19;25(3):e13443. doi: 10.1111/mpp.13443 (PMC10950013; doi:10.1111/mpp.13443)
Supplement: Supplementary file 1 — Figure S1. Phylogenetic analysis of Sso1 orthologues in eukaryotes. (A) A neighbour‐joining tree was constructed based on the amino acid sequences of the Sso1 orthologues. Numbers at the nodes represent the percentage of occurrence in 10,000 bootstrap replicates. (B) Domain structure of Sso1 orthologues in eukaryotes. The conserved syntaxin domains (blue) are shown. (C) Prediction of transmembrane helices in FocSso1 proteins. [file MPP-25-e13443-s013.pdf]

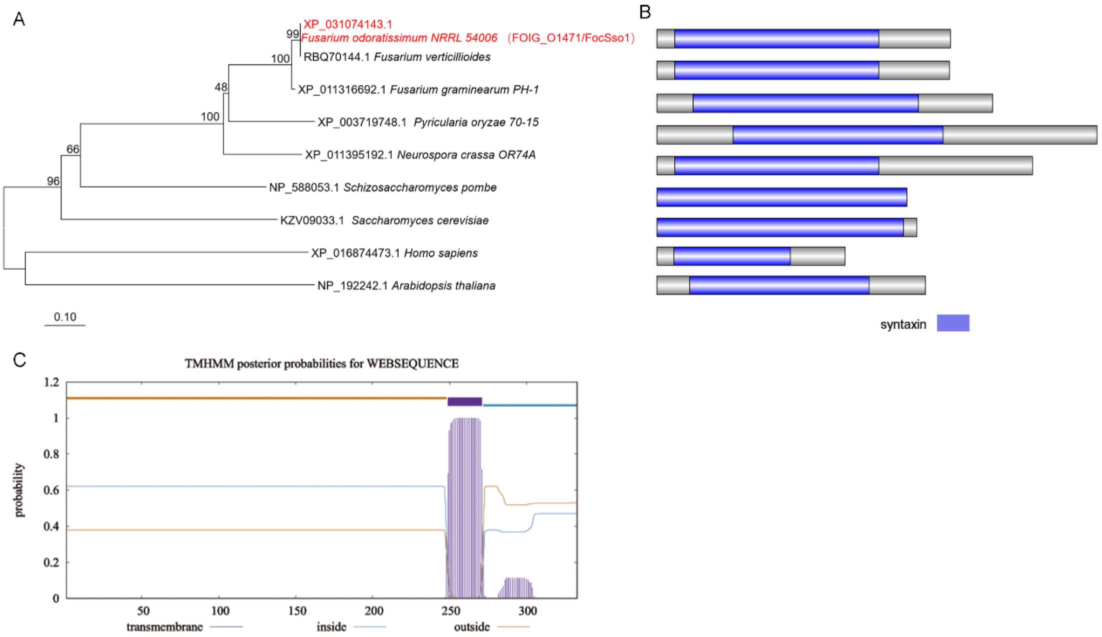

Fig. S1 Phylogenetic analysis of Sso1 orthologs in eukaryotes. (A) A neighbor-joining tree was constructed based on the amino acid sequences of the Sso1 orthologs. Numbers at the nodes represent the percentage of occurrence in 10,000 bootstrap replicates. (B) Domain structure of Sso1 orthologs in eukaryotes. The conserved syntaxin domains (blue) are shown. (C) Prediction of transmembrane helices in FocSso1 proteins.
