# Supplementary material for: Two distinct SNARE complexes mediate vesicle fusion with the plasma membrane to ensure effective development and pathogenesis of Fusarium oxysporum f. sp. cubense
Source: Mol Plant Pathol. 2024 Mar 19;25(3):e13443. doi: 10.1111/mpp.13443 (PMC10950013; doi:10.1111/mpp.13443)
Supplement: Supplementary file 2 — Figure S2. Gene deletion strategy and Southern blot assays. (A) Targeted gene‐replacement strategy for FocSSO1 is shown. The primer pairs F1 and R1, F3 and R3 were used to generate the gene replacement constructs. Primers F2 and R2 were used for mutant screening and identification. (B) Southern blot analysis for confirmation of gene deletions. NcoΙ‐digested genomic DNAs showed a 6.87 kb band in the wild type (WT) and a 3.81 kb band in the mutants. [file MPP-25-e13443-s011.pdf]

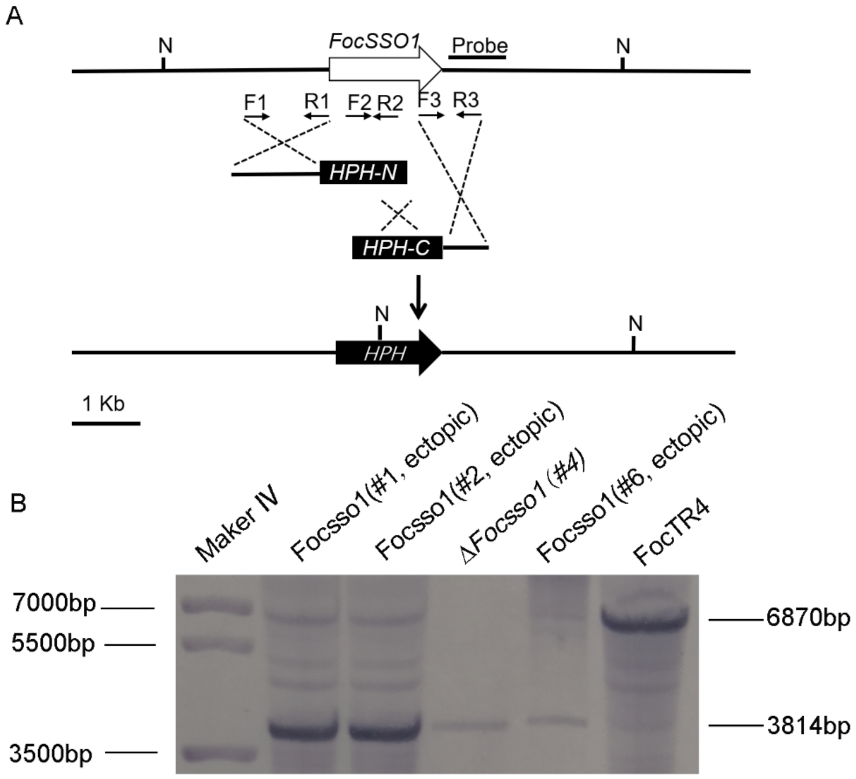

Fig. S2 Gene deletion strategy and Southern blot assays. (A) Targeted gene-replacement strategy for *FocSSO1* is shown. The primer pairs F1 and R1, F3 and R3 were used to generate the gene replacement constructs. Primers F2 and R2 were used for mutant screening and identification. (B) Southern blot analysis for confirmation of gene deletions. *Nco* I-digested genomic DNAs showed a 6.87 kb band in the WT and a 3.81 kb band in the mutants.
