# Supplementary material for: Two distinct SNARE complexes mediate vesicle fusion with the plasma membrane to ensure effective development and pathogenesis of Fusarium oxysporum f. sp. cubense
Source: Mol Plant Pathol. 2024 Mar 19;25(3):e13443. doi: 10.1111/mpp.13443 (PMC10950013; doi:10.1111/mpp.13443)
Supplement: Supplementary file 3 — Figure S3. Role of FocSso1 in the vegetative growth and sporulation of the Fusarium wilt fungus. (A) Colony morphology and growth of the wild type (FocTR4), ∆Focsso1 and ∆Focsso1‐C on complete medium (CM) and minimal medium (MM). (B) Graphical representation of the colony diameters of the indicated strains. (C) Number of microconidia produced by the indicated strains in potato dextrose broth. (D) Number of macroconidia produced by the indicated strains in Spezieller Nährstoffarmer agar. Values are presented as means ± SD (standard deviation) calculated from three independent experiments. **p < 0.05, ***p < 0.001. [file MPP-25-e13443-s015.pdf]

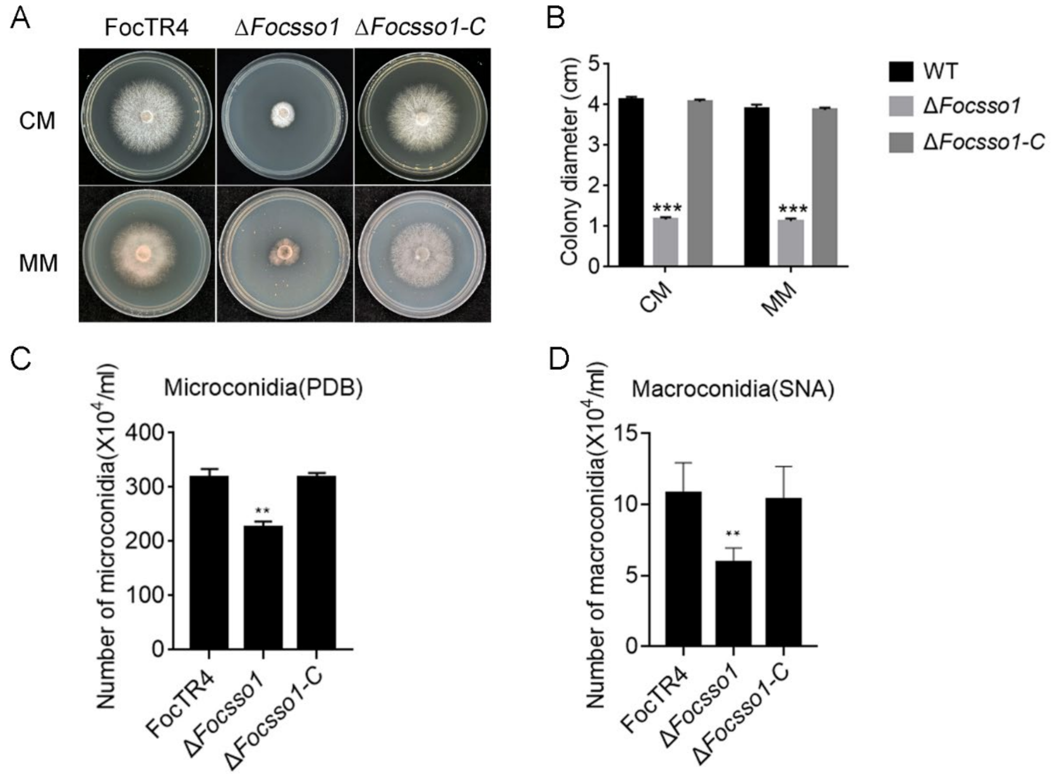

Fig. S3 Role of FocSso1 in the vegetative growth and sporulation of the fusarium wilt fungus. (A) Colony morphology and growth of the wild type (FocTR4), Δ*Focsso1* and Δ*Focsso1-C* on CM and MM media. (B) Graphical representation of the colony diameters of the indicated strains. (C) Number of microconidia produced by the indicated strains in PDB media. (D) Number of macroconidia produced by the indicated strains in SNA media. Values are presented as Means ± SD (standard deviation) calculated from three independent experiments. **, $P < 0.05$; ***, $P < 0.001$.
