# Supplementary material for: Two distinct SNARE complexes mediate vesicle fusion with the plasma membrane to ensure effective development and pathogenesis of Fusarium oxysporum f. sp. cubense
Source: Mol Plant Pathol. 2024 Mar 19;25(3):e13443. doi: 10.1111/mpp.13443 (PMC10950013; doi:10.1111/mpp.13443)
Supplement: Supplementary file 5 — Figure S5. Sensitivity of the wild‐type strain (FocTR4), FocSSO1 gene deletion mutant (ΔFocsso1) and complemented strain (ΔFocsso1‐C) to osmotic, oxidative and cell wall stresses. (A) Colonies of the indicated strains on complete medium (CM) supplemented with 0.02% (wt/vol) SDS, 0.7 M NaCl, 36 mM H2O2, 200 μg/mL Congo red (CR) and 200 μg/mL calcofluor white (CFW). (B) Mycelial radial growth inhibition rates were quantified 3 days after culturing the strains on CM with different stress‐inducing agents. **p < 0.05. [file MPP-25-e13443-s009.pdf]

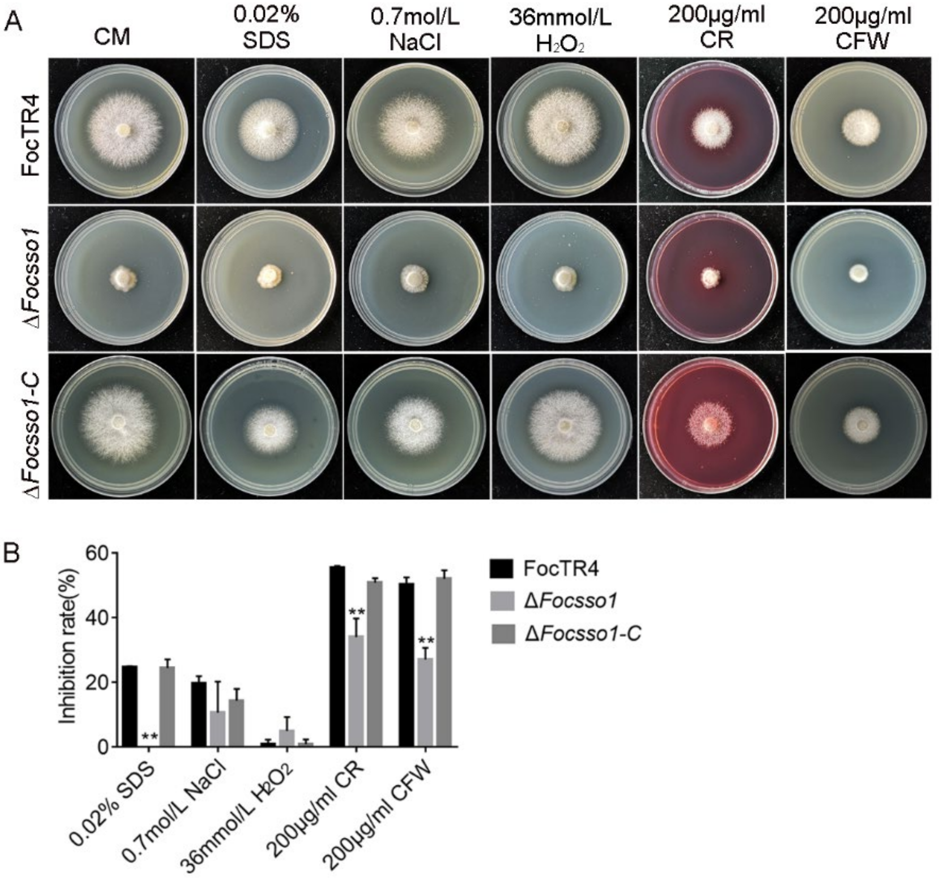

Fig. S5 Sensitivity of the wild-type strain (FocTR4), *FocSSO1* gene deletion mutant (Δ*Focsso1*) and complemented strain (Δ*Focsso1-C*) to osmotic, oxidative and cell wall stresses. (A) Colonies of the indicated strains on CM media supplemented with 0.02% (w/v) SDS, 0.7M NaCl, 36 mM H_2O_2, 200 μg/ml CR and 200 μg/ml CFW, respectively. (B) Mycelial radial growth inhibition rates were quantified 3 days after culturing the strains on CM media with different stress-inducing agents. **, $P < 0.05$.
