# Supplementary material for: Two distinct SNARE complexes mediate vesicle fusion with the plasma membrane to ensure effective development and pathogenesis of Fusarium oxysporum f. sp. cubense
Source: Mol Plant Pathol. 2024 Mar 19;25(3):e13443. doi: 10.1111/mpp.13443 (PMC10950013; doi:10.1111/mpp.13443)
Supplement: Supplementary file 6 — Figure S6. Identification of FocSso1 interacting proteins. (A) Number of identified proteins by affinity purification and mass spectrometry analysis in GFP‐FocSso1 and wild‐type (WT)‐GFP strains. (B) 705 proteins were specifically identified in GFP‐FocSso1 interactome but not in WT‐GFP. [file MPP-25-e13443-s008.pdf]

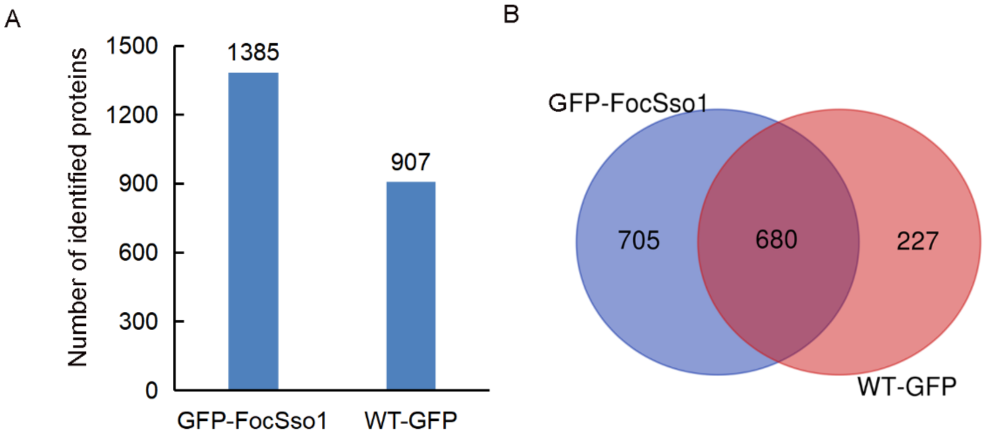

Fig. S6 Identification of FocSso1 interacting proteins. (A) Number of identified proteins by affinity purification and mass spectrometry analysis in GFP-FocSso1 and WT-GFP strains. (B) 705 proteins were specifically identified in GFP-FocSso1 interactome but not in WT-GFP.
