# Supplementary material for: Two distinct SNARE complexes mediate vesicle fusion with the plasma membrane to ensure effective development and pathogenesis of Fusarium oxysporum f. sp. cubense
Source: Mol Plant Pathol. 2024 Mar 19;25(3):e13443. doi: 10.1111/mpp.13443 (PMC10950013; doi:10.1111/mpp.13443)
Supplement: Supplementary file 7 — Figure S7. Phylogenetic analysis of Snc1, Sso2 and Sec9 orthologues in eukaryotes. (A) Phylogenetic tree and domain architectures of Snc1 orthologues in eukaryotes. (B) Phylogenetic tree and domain architectures of Sso2 orthologues in eukaryotes. (C) Phylogenetic tree and domain architectures of Sec9 orthologues in eukaryotes. [file MPP-25-e13443-s004.pdf]

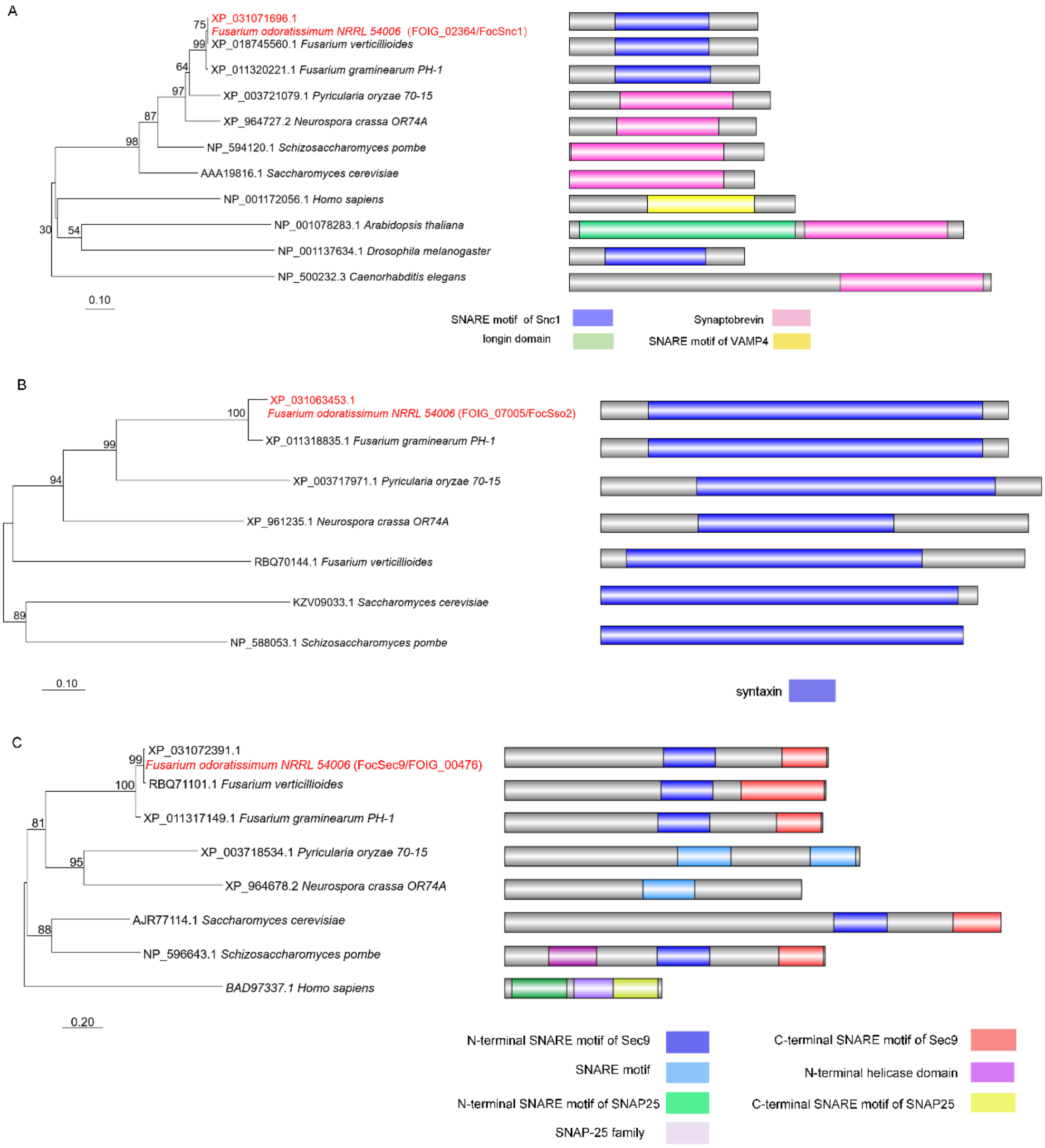


**Fig. S7 Phylogenetic analysis of Snc1, Sso2 and Sec9 orthologs in eukaryotes.** (A) Phylogenetic tree and domain architectures of Snc1 orthologs in eukaryotes. (B) Phylogenetic tree and domain architectures of Sso2 orthologs in eukaryotes. (C) Phylogenetic tree and domain architectures of Sec9 orthologs in eukaryotes.
