# Supplementary material for: Two distinct SNARE complexes mediate vesicle fusion with the plasma membrane to ensure effective development and pathogenesis of Fusarium oxysporum f. sp. cubense
Source: Mol Plant Pathol. 2024 Mar 19;25(3):e13443. doi: 10.1111/mpp.13443 (PMC10950013; doi:10.1111/mpp.13443)
Supplement: Supplementary file 8 — Figure S8. Targeted gene replacement strategy and Southern blot assays for FocSNC1 and FocSSO2 gene deletions. (A) Targeted gene replacement strategy for FocSNC1 is shown. NcoΙ‐digested genomic DNAs showed a 5.91 kb band in the wild type (WT) and a 1.88 kb band in the mutants. (B) The targeted gene‐replacement strategy for FocSSO2 is shown. NdeΙ‐digested genomic DNAs showed a 3.54 kb band in the WT and a 1.62 kb band in the mutants. [file MPP-25-e13443-s016.pdf]

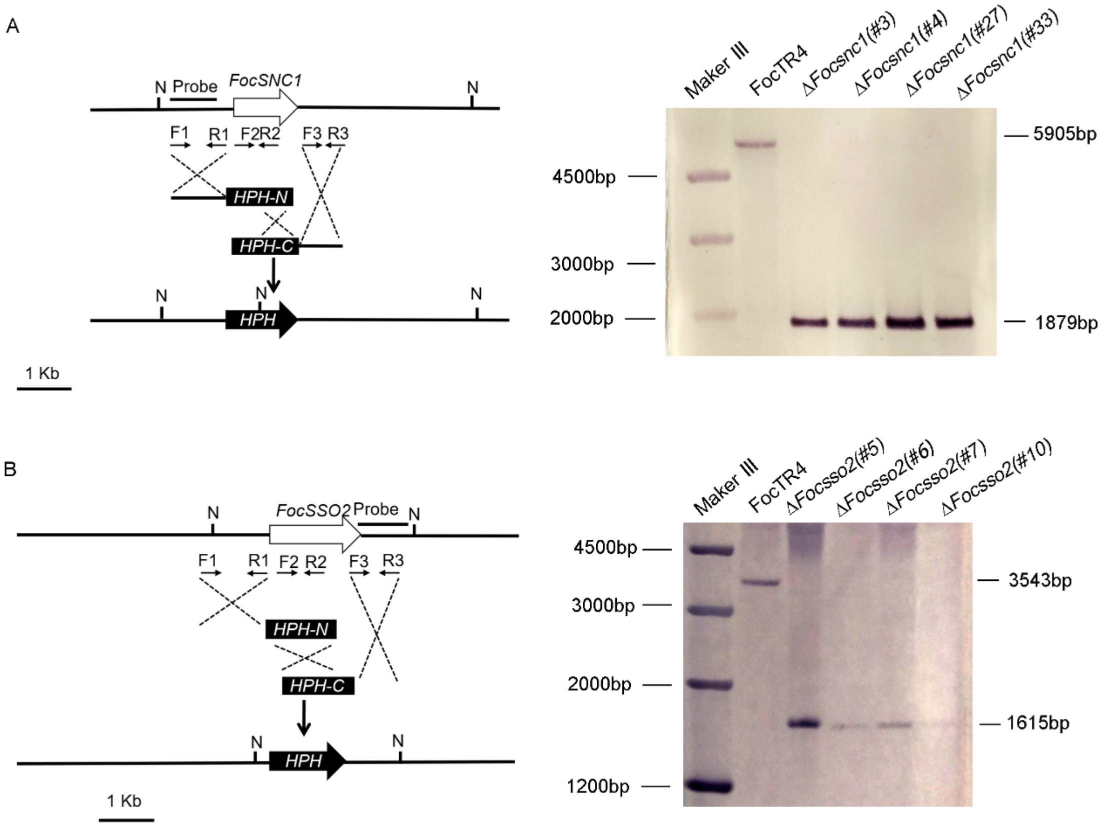

Fig. S8 Targeted gene replacement strategy and Southern blot assays for *FocSNC1* and *FocSSO2* gene deletions. (A) Targeted gene replacement strategy for *FocSNC1* is shown. *Nco* I-digested genomic DNAs showed a 5.91 kb band in the WT and a 1.88 kb band in the mutants. (B) The targeted gene-replacement strategy for *FocSSO2* is shown. *Nde* I-digested genomic DNAs showed a 3.54 kb band in the WT and a 1.62 kb band in the mutants.
