# Supplementary material for: Two distinct SNARE complexes mediate vesicle fusion with the plasma membrane to ensure effective development and pathogenesis of Fusarium oxysporum f. sp. cubense
Source: Mol Plant Pathol. 2024 Mar 19;25(3):e13443. doi: 10.1111/mpp.13443 (PMC10950013; doi:10.1111/mpp.13443)
Supplement: Supplementary file 9 — Figure S9. Phenotypic characterization of ∆Focsso2 and ∆Focsnc1 mutants. (A) Colony morphology and growth of the indicated strains on complete medium (CM) and minimal medium (MM). (B) Bar graph representation of the colony diameters of the indicated strains. (C) Number of microconidia produced by the indicated strains following growth in potato dextrose broth. (D) Number of macroconidia produced by the indicated strains following growth in Spezieller Nährstoffarmer agar. Values are presented as means ± SD calculated from three independent experiments. **p < 0.05, ***p < 0.001. [file MPP-25-e13443-s012.pdf]

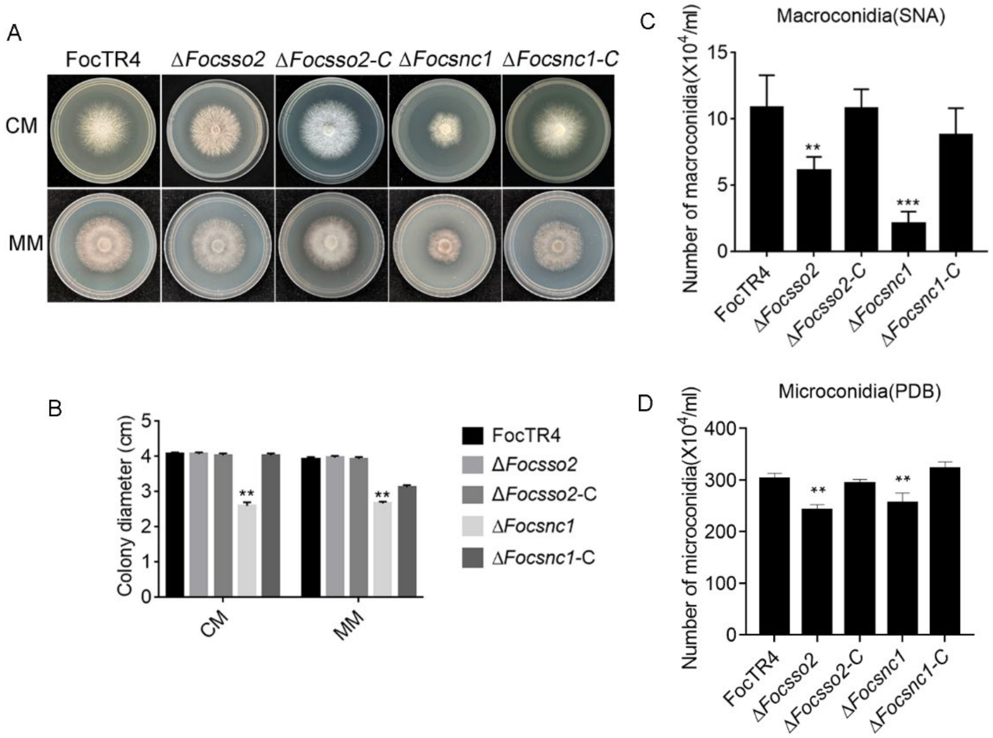

Fig. S9 Phenotypic characterization of Δ*Focsso2* and Δ*Focsnc1* mutants. (A) Colony morphology and growth of the indicated strains on CM and MM media. (B) Bar graph representation of the colony diameters of the indicated strains. (C) Number of microconidia produced by the indicated strains following growth in PDB media. (D) Number of macroconidia produced by the indicated strains following growth in SNA media. Values are presented as means ± SD (standard deviations) calculated from three independent experiments. **, $P < 0.05$; ***, $P < 0.001$.
