# Supplementary material for: Two distinct SNARE complexes mediate vesicle fusion with the plasma membrane to ensure effective development and pathogenesis of Fusarium oxysporum f. sp. cubense
Source: Mol Plant Pathol. 2024 Mar 19;25(3):e13443. doi: 10.1111/mpp.13443 (PMC10950013; doi:10.1111/mpp.13443)
Supplement: Supplementary file 10 — Figure S10. Sensitivity of the wild‐type strain (FocTR4), FocSSO2 gene deletion mutant (ΔFocsso2) and complemented strain (ΔFocsso2‐C) to osmotic, oxidative and cell wall stresses. (A) Colonies of the indicated strains on complete medium (CM) supplemented with 0.02% (wt/vol) SDS, 0.7 M NaCl, 36 mM H2O2, 200 μg/mL Congo red (CR) and 200 μg/mL calcofluor white (CFW). (B) Mycelial radial growth inhibition rates were quantified after culturing the strains on CM containing different stress‐inducing agents at 3 days post‐inoculation. [file MPP-25-e13443-s003.pdf]

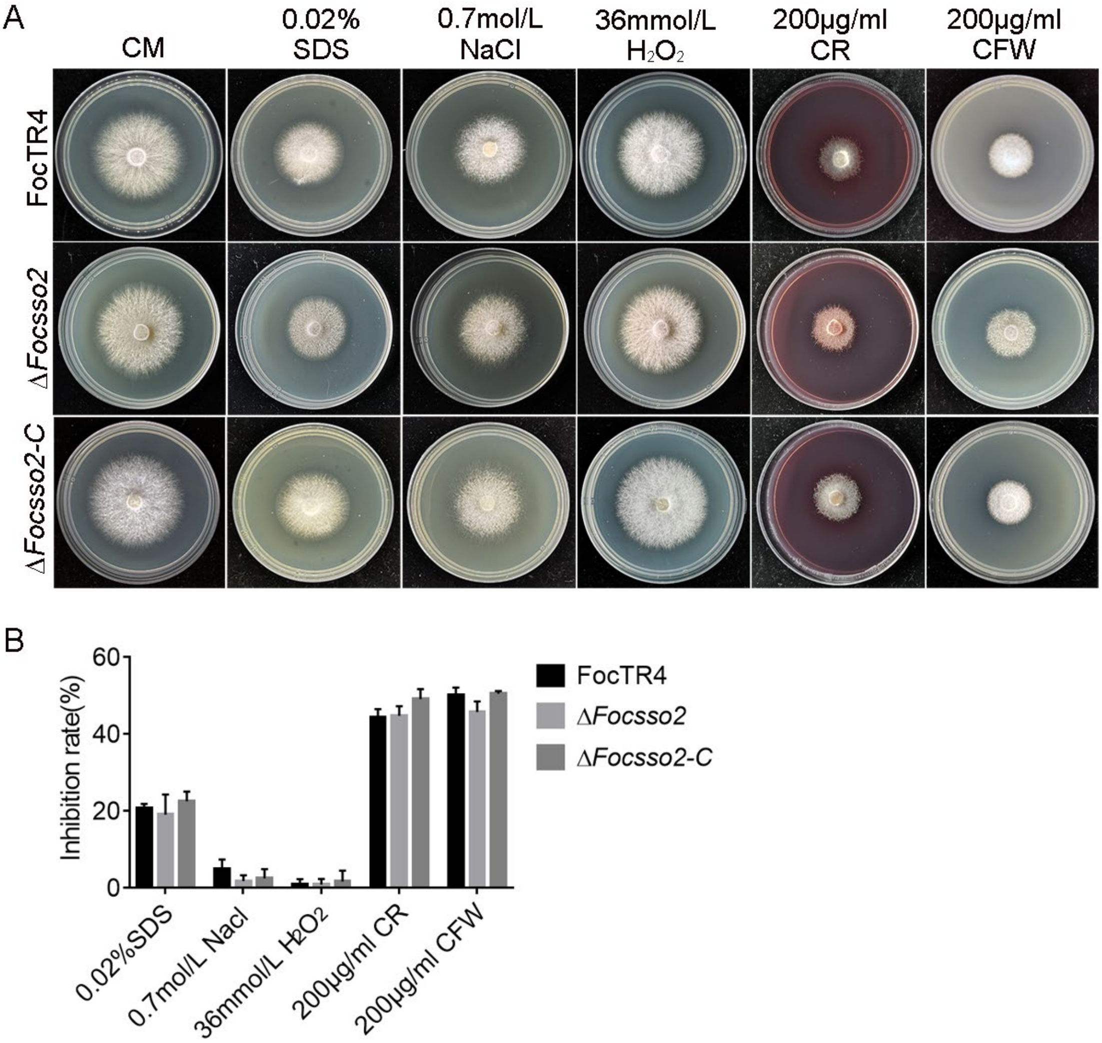

Fig. S10 Sensitivity of the wild-type strain (FocTR4), *FocSSO2* gene deletion mutant (Δ*Focsso2*) and complemented strain (Δ*Focsso2-C*) to osmotic, oxidative and cell wall stresses. (A) Colonies of the indicated strains on CM media supplemented with 0.02% (w/v) SDS, 0.7M NaCl, 36 mM H_2O_2, 200 µg/ml CR and 200 µg/ml CFW, respectively. (B) Mycelial radial growth inhibition rates were quantified after culturing the strains on CM media containing different stress-inducing agents at 3 days post-inoculation.
