# Supplementary material for: Two distinct SNARE complexes mediate vesicle fusion with the plasma membrane to ensure effective development and pathogenesis of Fusarium oxysporum f. sp. cubense
Source: Mol Plant Pathol. 2024 Mar 19;25(3):e13443. doi: 10.1111/mpp.13443 (PMC10950013; doi:10.1111/mpp.13443)
Supplement: Supplementary file 11 — Figure S11. Sensitivity of the wild‐type strain (FocTR4), FocSNC1 gene deletion mutant (ΔFocsnc1) and complemented strain (ΔFocsnc1‐C) to osmotic, oxidative and cell wall stresses. (A) Colonies of the indicated strains on complete medium (CM) supplemented with 0.02% (wt/vol) SDS, 0.7 M NaCl, 36 mM H2O2, 200 μg/mL Congo red (CR) and 200 μg/mL calcofluor white (CFW). (B) Mycelial radial growth inhibition rates were quantified after culturing the strains on CM containing different stress‐inducing agents at 3 days post‐inoculation. (C) Colonies of the indicated strains on CM supplemented with 0.7 M KCl and 0.7 M NaNO3. (D) Mycelial radial growth inhibition rates were quantified after culturing the strains on CM containing different stress‐inducing agents at 3 days post‐inoculation. **p < 0.05. [file MPP-25-e13443-s010.pdf]

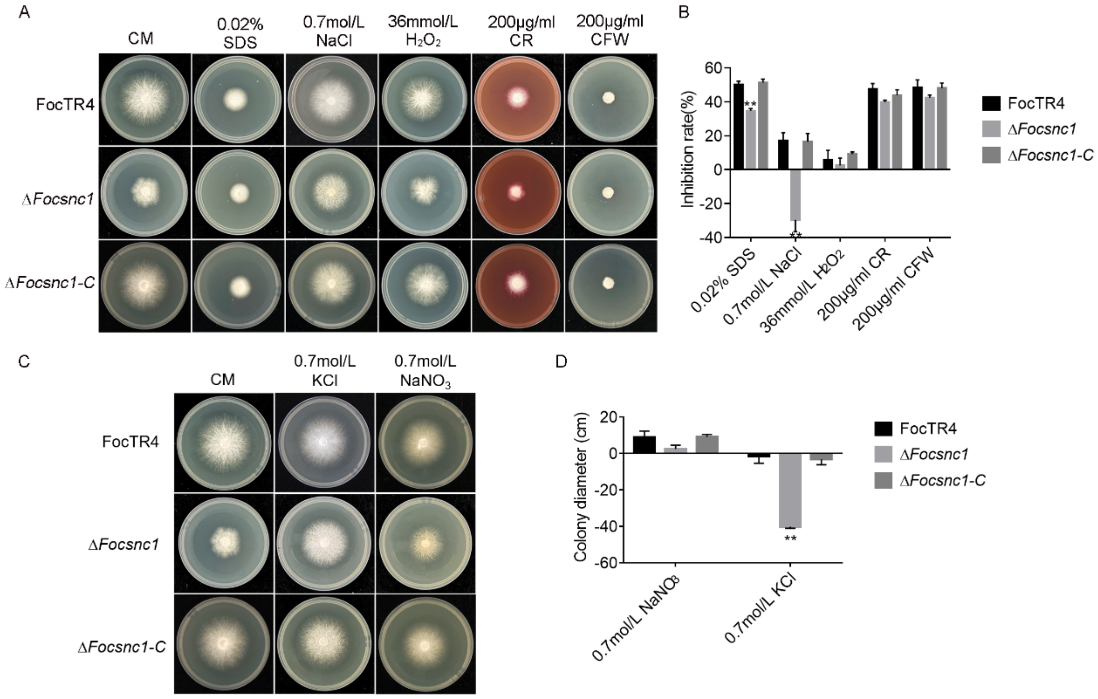

Fig. S11 Sensitivity of the wild type strain (FocTR4), *FocSNC1* gene deletion mutant (Δ*Focsnc1*) and complemented strain (Δ*Focsnc1-C*) to osmotic, oxidative and cell wall stresses. (A) Colonies of the indicated strains on CM media supplemented with 0.02% (w/v) SDS, 0.7M NaCl, 36 mM H_2O_2, 200 μg/ml CR and 200 μg/ml CFW, respectively. (B) Mycelial radial growth inhibition rates were quantified after culturing the strains on CM media containing different stress-inducing agents at 3 days post-inoculation. (C) Colonies of the indicated strains on CM media supplemented with 0.7M KCl and 0.7M $NaNO_3$, respectively. (D) Mycelial radial growth inhibition rates were quantified after culturing the strains on CM media containing different stress-inducing agents at 3 days post-inoculation. **, $P < 0.05$.
