# Supplementary material for: Two distinct SNARE complexes mediate vesicle fusion with the plasma membrane to ensure effective development and pathogenesis of Fusarium oxysporum f. sp. cubense
Source: Mol Plant Pathol. 2024 Mar 19;25(3):e13443. doi: 10.1111/mpp.13443 (PMC10950013; doi:10.1111/mpp.13443)
Supplement: Supplementary file 12 — Figure S12. Continuous movement of GFP‐FocSnc1 to hyphal apex and plasma membrane. (A) Representative time series images of GFP‐FocSnc1 on the hyphal apex immediately after photobleach (t = 0 s). Fluorescence recovery after photobleaching (FRAP) recovery in the photobleaching site after 1 min. (B) Representative time series images of GFP‐FocSnc1 on the plasma membrane of immediately following the photobleach (t = 0 s). Fluorescence recovery after photobleaching (FRAP) at the bleaching site after 1 min. Bar, 10 μm. [file MPP-25-e13443-s005.pdf]

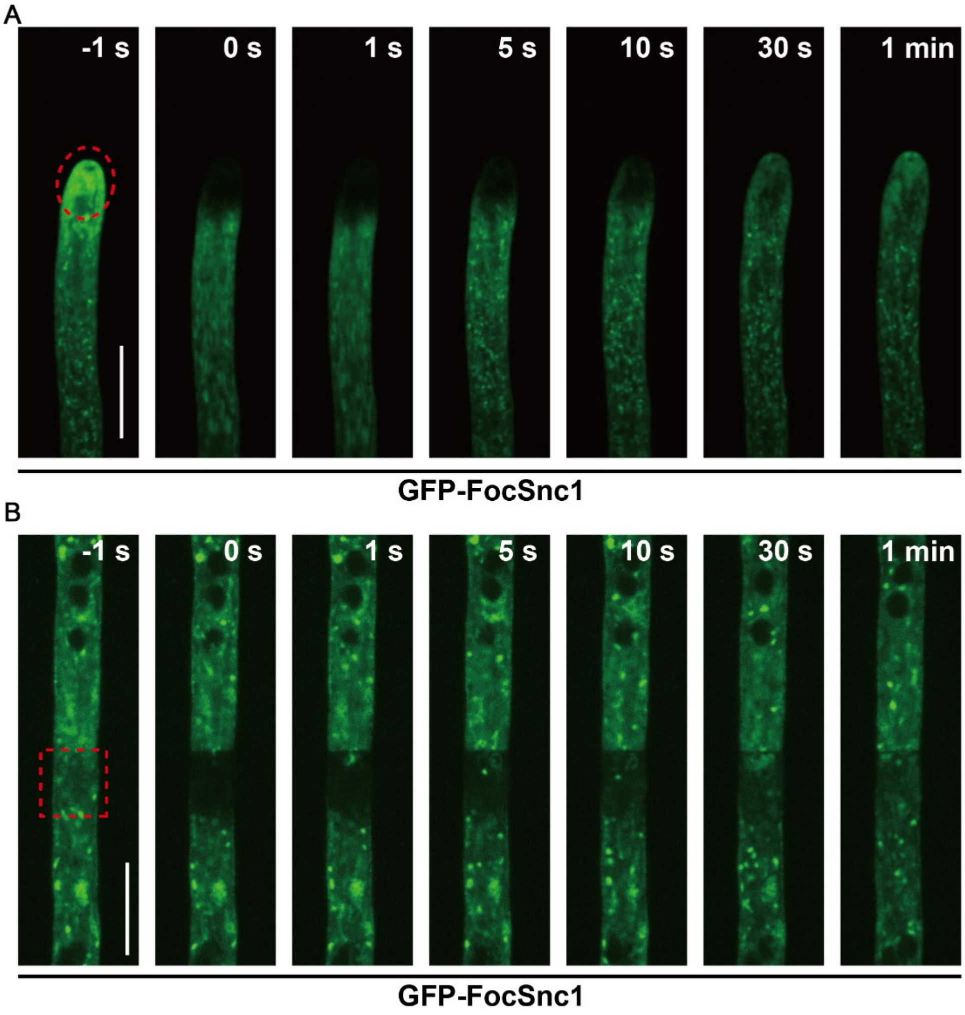

Fig. S12 Continuous movement of GFP-FocSnc1 to hyphal apex and plasma membrane. (A) Representative time series images of GFP-FocSnc1 on the hyphal apex immediately after photobleach (t= 0 s). Fluorescence recovery after photobleaching (FRAP) recovery in the photobleaching site after 1 min. (B) Representative time series images of GFP-FocSnc1 on the plasma membrane of immediately following the photobleach (t= 0 s). Fluorescence recovery after photobleaching (FRAP) at the bleaching site after 1 min. Bar, 10 μm.
