# Supplementary material for: Two distinct SNARE complexes mediate vesicle fusion with the plasma membrane to ensure effective development and pathogenesis of Fusarium oxysporum f. sp. cubense
Source: Mol Plant Pathol. 2024 Mar 19;25(3):e13443. doi: 10.1111/mpp.13443 (PMC10950013; doi:10.1111/mpp.13443)
Supplement: Supplementary file 14 — Table S2. Wild‐type and mutant strains of fungi used in this study. [file MPP-25-e13443-s006.pdf]

Table S2 Wild-type and mutant strains of fungi used in this study

Strain	Genotype description	Reference
FocTR4	Wild-type	Yun et al., 2019
ΔFocsso1	*Focsso1* deletion mutant of FocTR4	This study
ΔFocsnc1	*Focsnc1* deletion mutant of FocTR4	This study
ΔFocsso2	*Focsso2* deletion mutant of FocTR4	This study
ΔFocsso1-C	*ΔFocsso1* transformant expressing GFP-FocSso1 construct	This study
ΔFocsnc1-C	*ΔFocsnc1* transformant expressing GFP-FocSnc1 construct	This study
ΔFocsso2-C	*ΔFocsso2* transformant expressing GFP-FocSso2 construct	This study
ΔFocsso1+GFP-FocSso2	*ΔFocsso1* transformant expressing GFP-FocSso2 construct	This study
ΔFocsso1+GFP-FocSnc1	*ΔFocsso1* transformant expressing GFP-FocSnc1 construct	This study
ΔFocsso2+GFP-FocSso1	*ΔFocsso2* transformant expressing GFP-FocSso1 construct	This study
ΔFocsso2+GFP-FocSnc1	*ΔFocsso2* transformant expressing GFP-FocSnc1 construct	This study
ΔFocsnc1+GFP-FocSso1	*ΔFocsnc1* transformant expressing GFP-FocSso1 construct	This study
ΔFocsnc1+GFP-FocSso2	*ΔFocsnc1* transformant expressing GFP-FocSso2 construct	This study
FocTR4+RP27-GFP	FocTR4 transformant expressing GFP construct	This study
ΔFocsso1+RP27-GFP	*ΔFocsso1* transformant expressing GFP construct	This study
ΔFocsso2+RP27-GFP	*ΔFocsso2* transformant expressing GFP construct	This study
ΔFocsnc1+RP27-GFP	*ΔFocsnc1* transformant expressing GFP construct	This study
GFP-FocSso1+Myc-	FocTR4 transformant expressing both GFP-	This study

FocSec9	FocSso1 and Myc-FocSec9 constructs	
GFP-FocSso2+Myc-FocSec9	FocTR4 transformant expressing both GFP-FocSso2 and Myc-FocSec9 constructs	This study
GFP-FocSnc1+Myc-FocSec9	FocTR4 transformant expressing both GFP-FocSnc1 and Myc-FocSec9 constructs	This study
GFP-FocSso2+Myc-FocSso1	FocTR4 transformant expressing both GFP-FocSso2 and Myc-FocSso1constructs	This study
GFP-FocSnc1+Myc-FocSso1	FocTR4 transformant expressing both GFP-FocSnc1 and Myc-FocSso1 constructs	This study
GFP-FocSnc1+Myc-FocSso2	FocTR4 transformant expressing both GFP-FocSnc1 and Myc-FocSso2 constructs	This study

Yun, Y., Song, A., Bao, J., Chen, S., Lu, S., Cheng, C., et al. (2019) Genome Data of Fusarium oxysporum f. sp. cubense race 1 and tropical race 4 isolates using long-read sequencing. Molecular Plant–Microbe Interactions, 32, 1270–1272.
