# Supplementary material for: Two distinct SNARE complexes mediate vesicle fusion with the plasma membrane to ensure effective development and pathogenesis of Fusarium oxysporum f. sp. cubense
Source: Mol Plant Pathol. 2024 Mar 19;25(3):e13443. doi: 10.1111/mpp.13443 (PMC10950013; doi:10.1111/mpp.13443)
Supplement: Supplementary file 15 — Table S3. PCR primers used in this study. [file MPP-25-e13443-s014.pdf]

Table S3 PCR primers used in this study

Primers	Sequence(5'-3')	Application
FOIG_01471-AF	CTGGACAAAATAAGATAGCGAG	*FocSSO1* deletion and probe
FOIG_01471-AR	TTGACCTCCACTAGCTCCAGCCAAGCCGCAATAAC TCAGGCAAAACATA	
FOIG_01471-BF	GAATAGAGTAGATGCCGACCGCGGGTTCCGACAAC ACAGCATCCTCT	
FOIG_01471-BR	AAGAGGCGTGTGGTAATAGG	
FOIG_01471-OF	ACTCACTTTCCGCTGATACCAT	Δ*Focsso1* mutant screen
FOIG_01471-OR	ACATACCACAGATACCCAAGCA	
FOIG_01471-UA	ACCACGAATAACGAATAGCC	
H853	AACTCACCGCGACGTCTGTC	
FOIG_07005-AF	AATGGCACCTCACGCAAT	*FocSSO2* deletion and probe
FOIG_07005-AR	TTGACCTCCACTAGCTCCAGCCAAGCCGGATTTTG AGCACGGGTC	
FOIG_07005-BF	GAATAGAGTAGATGCCGACCGCGGGTTTTGTTCCT CGTTTTCCCTTC	
FOIG_07005-BR	TTGATTTAGGATGGGATGCG	
FOIG_07005-OF	AGGATTATTCGCAACAGGCT	Δ*Focsso2* mutant screen
FOIG_07005-OR	CAATAGCGATGACGATGAGC	
FOIG_07005-UA	GCACGGTCCAACTGTCTCAT	
FOIG_02364-AF	TGTATCTGCCTCTTGCGTGG	*FocSNC1* deletion and probe
FOIG_02364-AR	TTGACCTCCACTAGCTCCAGCCAAGCCGTCGTGTC GTGTCGTTGTGA	
FOIG_02364-BF	GAATAGAGTAGATGCCGACCGCGGGTTGGTCCTTA	

	CGATACACACGC	
FOIG_02364-BR	ACTACCAAATGACGCCAACA	
FOIG_02364-OF	TTACGACCCCTATATCCCCA	Δ*Focsnc1* mutant screen
FOIG_02364-OR	ATCCTTCCACCACATCTGCT	
FOIG_02364-UA	CGACCCCTGTTATCAGTTTT	
GFP-F	ATGGTGAGCAAGGGCGAGGA	Used to expand GFP
GFP-R	CTTGTACAGCTCGTCCATGC	
FocSSO1-PF	GGGTACCGGGCCCCCCCTCGAGGATATCCTGGCTTCTCTGACA	Δ*Focsso1* complementation and localization
FocSSO1-PR	TCCTCGCCCTTGCTCACCATCTCGACGCTACCTATCCCTCA	
FocSSO1-OF	GCATGGACGAGCTGTACAAGATGGCTCCCCTTGCCCACAAC	
FocSSO1-OR	CGACCTGCAGGCATGCAAGCTTTAAAGAGCTCCTTCCGTCATC	
FocSSO2-PF	GGGTACCGGGCCCCCCCTCGAGACACGAACCGCCAAGAAG	Δ*Focsso2* complementation and localization
FocSSO2-PR	TCCTCGCCCTTGCTCACCATGGTGATCAATTATACTCGAAC	
FocSSO2-OF	GCATGGACGAGCTGTACAAGATGTCCTACGATCAGTACAA	
FocSSO2-OR	CGACCTGCAGGCATGCAAGCTTAACATCATAACAGCGGCAAT	
FocSNC1-PF	GGGTACCGGGCCCCCCCTCGAGTTCGCCGCGTCGTCGACAAG	Δ*Focsnc1* complementation and localization
FocSNC1-PR	TCCTCGCCCTTGCTCACCATTTTGGCTGTGGTTTGGAGAT	
FocSNC1-OF	GCATGGACGAGCTGTACAAGATGCCTGAGCAGGAAGCCCC	
FocSNC1-OR	CGACCTGCAGGCATGCAAGCTTCCGTTGCATCATGATAGAAT	
FocSec9pBT3-STE-F	ATTAACAAGGCCATTACGGCCATGAAGAAGTTTGGCTTTGG	For generation of pFocSec9-pBT3-STE constructs
FocSec9pBT3-STE-R	AACTGATTGGCCGAGGCGGCCCCCTTGATACGGGCCAATCGTT	
FocSso1pBT3-N-F	ATTAACAAGGCCATTACGGCCATGGCTCCCCTTGCCCACAA	For generation of pFocSso1-pBT3-N constructs
FocSso1pBT3-N-	AACTGATTGGCCGAGGCGGCCGTTCCGCAATAGCT	

R	GAGGTA	
FocSso2pBT3-N-F	ATTAACAAGGCCATTACGGCCATGTCCTACGATCAGTACAA	For generation of pFocSso2-pBT3-N constructs
FocSso2pBT3-N-R	AACTGATTGGCCGAGGCGGCCCTTGTTGTTGTTGGCCACAG	
FocSso1-pPR3-N-F	CAGAGTGGCCATTACGGCCCATGGCTCCCCTTGCCCACAA	For generation of pFocSso1-pPR3-N constructs
FocSso1-pPR3-N-R	TCGAGAGGCCGAGGCGGCCGTTCCGCAATAGCTGAGGTA	
FocSso2-pPR3-N-F	CAGAGTGGCCATTACGGCCCATGTCCTACGATCAGTACAA	For generation of pFocSso2-pPR3-N constructs
FocSso2-pPR3-N-R	TCGAGAGGCCGAGGCGGCCCTTGTTGTTGTTGGCCACAG	
FocSnc1-pPR3-N-F	CAGAGTGGCCATTACGGCCCATGCCTGAGCAGGAAGCCCC	For generation of pFocSnc1-pPR3-N constructs
FocSnc1-pPR3-N-R	TCGAGAGGCCGAGGCGGCCACGAGTAGCCACAACAGAGG	
FocSEC9PF-MYC	AGGGAACAAAAGCTGGGTACCGCGGTCAGTTGCCAGCTCAG	For generation of pMYC-FocSec9 construct
FocSEC9PR-MYC	CAGGTCCTCCTCTGAGATCAGCTTCTGCTCCATGGCAAGATTTGTTGACTGTG	
FocSEC9OF-MYC	TGGAGCAGAAGCTGATCTCAGAGGAGGACCTGAAGAAGTTTGGCTTTGGAAA	
FocSEC9OR-MYC	GCCGAATTCGATATCAAGCTTATCAATGAATGCGAAGTGTT	
FocSso1PF-MYC	AGGGAACAAAAGCTGGGTACCGATATCCTGGCTTCTCTGAC	For generation of pMYC-FocSso1 construct
FocSso1PR-MYC	CAGGTCCTCCTCTGAGATCAGCTTCTGCTCCATCTCGACGCTACCTATCCCTC	
FocSso1OF-MYC	ATGGAGCAGAAGCTGATCTCAGAGGAGGACCTGGCTCCCCTTGCCCACAACGC	
FocSso1OR-MYC	GCCGAATTCGATATCAAGCTTTAAAGAGCTCCTTCCGTCATC	
FocSso2PF-MYC	AGGGAACAAAAGCTGGGTACCACACGAACCGCCAAGAAG	For generation of pMYC-FocSso2 construct
FocSso2PR-MYC	CAGGTCCTCCTCTGAGATCAGCTTCTGCTCCATGGTGATCAATTATACTCGAAC	
FocSso2OF-	ATGGAGCAGAAGCTGATCTCAGAGGAGGACCTGTC	

MYC	CTACGATCAGTACAATCA	
FocSso2OR-MYC	GCCGAATTCGATATCAAGCTTAACATCATAACAGCGGCAAT	
