# Supplementary material for: Two distinct SNARE complexes mediate vesicle fusion with the plasma membrane to ensure effective development and pathogenesis of Fusarium oxysporum f. sp. cubense
Source: Mol Plant Pathol. 2024 Mar 19;25(3):e13443. doi: 10.1111/mpp.13443 (PMC10950013; doi:10.1111/mpp.13443)
Supplement: Supplementary file 16 — Table S4. Plasmids used in this study. [file MPP-25-e13443-s018.pdf]

Table S4. Plasmids used in this study

Clone	Description
pGFP-FocSso1	For expression of GFP:FocSso1, cloned in XhoI-*Hind*III sites of pKNT. Ampicillin and Neomycin resistance.
pGFP-FocSso2	For expression of GFP:FocSso2, cloned in XhoI-*Hind*III sites of pKNT. Ampicillin and Neomycin resistance.
pGFP-FocSnc1	For expression of GFP:FocSnc1, cloned in XhoI-*Hind*III sites of pKNT. Ampicillin and Neomycin resistance.
pFocSso1-pBT3-N	FocSso1 was cloned in pBT3-N. Kanamycin resistance.
pFocSso2-pBT3-N	FocSso2 was cloned in pBT3-N. Kanamycin resistance.
pFocSec9-pBT3-STE	FocSec9 was cloned in pBT3-STE. Kanamycin resistance.
pFocSso1-pPR3-N	FocSso1 was cloned in pPR3-N. Ampicillin resistance.
pFocSso2-pPR3-N	FocSso2 was cloned in pPR3-N. Ampicillin resistance.
pFocSnc1-pPR3-N	FocSnc1 was cloned in pPR3-N. Ampicillin resistance.
pBT3-N	Original bait plasmid from DUALhunter starter kit.
pPR3-N	Original prey plasmid from DUALhunter starter kit.
pOst-NubI	Functional control plasmid from DUALhunter starter kit.
pTSU2-APP	Positive control bait plasmid from DUALhunter starter kit.
pNubG-Fe65	Positive control prey plasmid from DUALhunter starter kit.
pMYC-FocSso1	For expression of Myc:FocSso1, cloned in *Kpn*I-*Hind*III sites of pKNT. Ampicillin and Hygromycin resistance.
pMYC-FocSso2	For expression of Myc:FocSso2, cloned in *Kpn*I-*Hind*III sites of pKNT. Ampicillin and Hygromycin resistance.
pMYC-FocSec9	For expression of Myc:FocSec9, cloned in *Kpn*I-*Hind*III sites of pKNT. Ampicillin and Hygromycin resistance.
